# Regio-Specific *N*-Glycome and *N*-Glycoproteome Map of the Elderly Human Brain With and Without Alzheimer’s Disease

**DOI:** 10.1016/j.mcpro.2022.100427

**Published:** 2022-10-14

**Authors:** Jennyfer Tena, Izumi Maezawa, Mariana Barboza, Maurice Wong, Chenghao Zhu, Michael Russelle Alvarez, Lee-Way Jin, Angela M. Zivkovic, Carlito B. Lebrilla

**Affiliations:** 1 Department of Chemistry, University of California, Davis, California, USA; 2 Department of Pathology and Laboratory Medicine, School of Medicine, University of California, Davis, Sacramento, California, USA; 3 UC Davis MIND Institute, Sacramento, California, USA; 4 Department of Anatomy, Physiology and Cell Biology, School of Veterinary Medicine, University of California Davis, Davis, California, USA; 5 Department of Nutrition, University of California, Davis, Davis, California, USA; 6 Institute of Chemistry, University of the Philippines Los Baños, Laguna, Philippines

**Keywords:** LC–MS, glycosylation, brain glycosylation, glycoproteins, human brain, Alzheimer's Disease, brain map, AD, Alzheimer’s disease, AT1B1, ATPase subunit beta-1, AT1B2, ATPase subunit beta-2, AT1B3, ATPase subunit beta-3, HS, hippocampal sclerosis, MF, membrane fraction, NCAM, neural cell adhesion molecule, NCI, no cognitive impairment

## Abstract

The proteins in the cell membrane of the brain are modified by glycans in highly interactive regions. The glycans and glycoproteins are involved in cell–cell interactions that are of fundamental importance to the brain. In this study, the comprehensive N-glycome and N-glycoproteome of the brain were determined in 11 functional brain regions, some of them known to be affected with the progression of Alzheimer’s disease. N-glycans throughout the regions were generally highly branched and highly sialofucosylated. Regional variations were also found with regard to the glycan types including high mannose and complex-type structures. Glycoproteomic analysis identified the proteins that differed in glycosylation in the various regions. To obtain the broader representation of glycan compositions, four subjects with two in their 70s and two in their 90s representing two Alzheimer's disease subjects, one hippocampal sclerosis subject, and one subject with no cognitive impairment were analyzed. The four subjects were all glycomically mapped across 11 brain regions. Marked differences in the glycomic and glycoproteomic profiles were observed between the samples.

## Highlights

• The *N*-glycome and *N*-glycoproteome map of the human brain is characterized.
• A liquid chromatography-mass spectrometry platform is successfully established.
• Region-specific glycosylation of 11 functional human brain regions is examined.
• Site-specific glycosylation of glycoproteins shows widespread changes.

## Graphical Abstract

**Figure:**
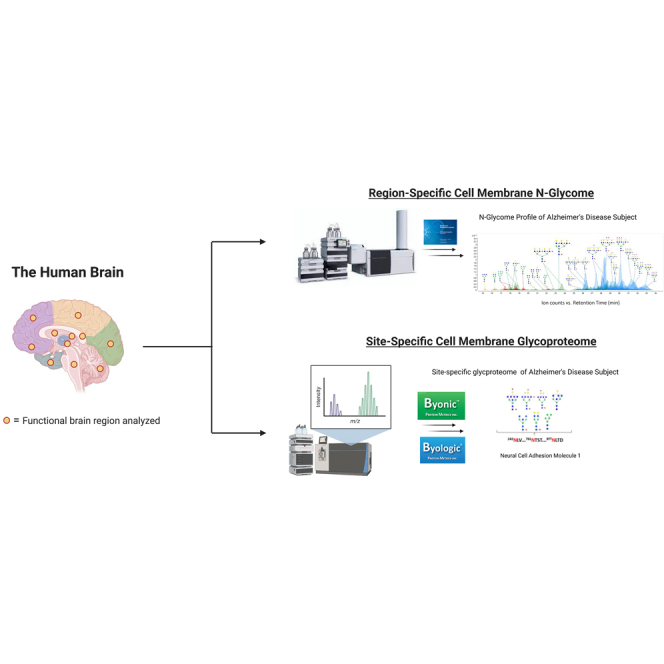

## In Brief

Glycosylation plays an important role in brain development and function. The cell membrane–associated N-glycans and glycoproteins from the human brain were characterized using a nanoflow LC–MS/MS platform. The brain regions included the frontal, temporal, parietal, occipital, cingulate, lateral cerebellar and orbitofrontal cortex, posterior hippocampus, thalamus, caudate nucleus, and pons. We illustrate the utility of glycomic and glycoproteomic tools to define the general breadth and depth in glycans associated with the elderly human brain and some neuropathological conditions.

Glycans on proteins create an extensive interactive network on the cell surface known as the glycocalyx. In the brain and other tissues, they are found in the extracellular matrix facilitating cell–cell and cell–matrix interactions resulting in intercellular signaling and adhesion (1, 2, 3). While glycosylation is indeed a common post-translational modification of proteins, they are also the most structurally complicated. Until recently, the limitations in our ability to determine and quantitate structures have challenged our ability to obtain a deeper understanding of the role of glycosylation. Unlike the genome and proteome, there is no template for the creation of the glycome (the totality of glycan structures). Glycans are metabolically synthesized to yield a suite of structures that vary heterogeneously by linkage, length, number of antennae, and composition.

The glycocalyx plays a crucial role in maintaining brain homeostasis (4). Protein N-glycosylation is a specific type of glycosylation where N-glycans are covalently attached to an asparagine by an N-glycosidic bond. N-glycosylation is also the most abundant type of glycosylation, with approximately 90% of all eukaryotic cells carrying N-glycosylated glycoproteins (5). The crucial role of N-glycosylation is highlighted in the central nervous system where alterations in N-glycosylation have been linked to different neuropathological symptoms such as mental retardation and epilepsy (6). As a result, N-glycans and N-glycoproteins make compelling targets for therapeutics and as biomarkers for diseases. Although the importance of N-glycans for neural development has been documented (7), understanding how the N-glycome is altered in response to a disease state is of importance and remains to be thoroughly studied. Most efforts have focused specifically on cancer (8) where glycosylation has been shown to be altered during cancer progression and metastasis (9). Similarly, in brain cancer, aberrant alterations in glycosylation have been reported pertaining to sialylation and fucosylation of N-glycans (10).

Recent studies have indicated glycomic profiles of the mammalian brain in neurological disorders such as Alzheimer’s disease (AD) and Parkinson’s disease (11, 12, 13). AD is an irreversible neurodegenerative disorder characterized by the loss of neurons in a region-specific manner particularly in the cortex and hippocampus. Nearly all the important brain proteins associated with AD pathogenesis are glycosylated (14). Changes in glycosylation for several key proteins, including amyloid precursor protein (15), β-site amyloid precursor protein–cleaving enzyme (16), and tau (17), among others, have been reported. The glycosylation changes observed on the key proteins involved sialylation and the presence of bisecting GlcNAc (16, 18, 19). In Parkinson’s disease, which is characterized by the progressive loss of functional dopaminergic neurons, changes in the region- and age-specific N-glycan compositional profiles have also been observed (20).

A comprehensive glycan map of the human brain representing various functional regions can yield a better understanding of the roles of glycans as well as provide potential therapeutic targets for brain diseases. Presently, studies on brain glycosylation have focused on limited structural features with even more limited regions. The efforts have employed primarily lectins, which are glycan-recognizing proteins on selected brain tissues (11). More structurally, intensive analyses have been performed using LC–MS (21). In a recent study, glycan profiles of nine regions of the mouse brain and a single region of human brain across different ages were examined (21). In general, human brain studies have focused on the brain as a whole (22) or a very limited number such as two (23). Rodent brains, which are more accessible, have been more widely studied in terms of their spatial and temporal variations (24, 25, 26). However, to our best knowledge, there have been no reported glycomic profiling of the elderly human brain.

Neurodegenerative disorders such as AD have an estimated prevalence of 10 to 30% in the population of 65 years and older (27). The brain regions first affected include the frontal and temporal lobes and then slowly progresses to impact other areas of the neocortex. To study the glycosylation of the brain of elderly patients, we determined the extent of glycan variations associated with various brain regions. In this report, we describe a limited pilot study to examine the variation in the N-glycome and N-glycoproteome of postmortem human brain tissues from four different individuals across 11 functional brain regions. The cell membrane–associated N-glycans and N-glycoproteins from the regions were determined using nanoflow LC–MS/MS platform (28). The brain regions included the frontal, temporal, parietal, occipital, cingulate, lateral cerebellar and orbitofrontal cortex, posterior hippocampus, thalamus, caudate nucleus, and pons. Two subjects in their 70s and two in their 90s with confirmed neurodegenerative diagnosis were examined with the goal of illustrating the utility of glycomic and glycoproteomic tools and to define the general breadth of the variations in glycans associated with the elderly brain and some neuropathological conditions.

## Experimental Procedures

### Experimental Design

The samples included postmortem tissues from two sets of donors: two males in their 70s and two males in their 90s. The subjects were chosen to represent two disease pathologies. Among the 70 year olds, one had pathologically confirmed AD (AD-74), and the other had no cognitive impairment (NCI-72) (supplemental Table S1). Among the 90 year olds, one had pathology confirmed AD (AD-93), and the other was diagnosed with hippocampal sclerosis (HS-95) or a subtype of neuropathological changes of limbic-predominant age-related TDP-43 encephalopathy (LATE-NC + HS). The brain from the four subjects was separated into 11 functional regions including the frontal, temporal, parietal, occipital, cingulate, lateral cerebellar and orbitofrontal cortex, posterior hippocampus, thalamus, caudate nucleus, and pons as shown in supplemental Table S1. From these regions, the cell membrane fractions (MFs) were enriched, processed, and analyzed for the glycome and glycoproteome.

### Cell Membrane Extraction From Human Brain Tissue

Human postmortem brain samples were drawn from the brain repositories of the University of California Davis Alzheimer’s Disease Research Center (P30-AG072972). The studies in this work abide by the Declaration of Helsinki principles. Written informed consent including consent for autopsy was obtained from study participants or for those with substantial cognitive impairment, a caregiver, legal guardian, or other proxy. Study protocols were reviewed and approved by the Institutional Review Board. For postmortem diagnosis, we followed the National Institute on Aging-Alzheimer’s Association guideline for the neuropathologic assessment of AD (29). The brain tissue used for the current study was snap frozen during autopsy and was stored in −80 °C until use.

For the glycomic analysis, both gray matter and white matter were combined and processed. Tissue samples were homogenized and resuspended in homogenization buffer containing 0.25 M sucrose, 20 mM Hepes–KOH (pH 7.4), and a 1:100 protease inhibitor cocktail set V, EDTA-free (Calbiochem; catalog no.: 539137). Cells were lysed on ice using a probe sonicator operated with alternating on and off pulses of 5 and 10 s, respectively. Lysates were pelleted by centrifugation at 9000*g* for 10 min to remove the nuclear fraction and cell debris. The supernatant was transferred to high-speed tubes, loaded onto a Beckman Optima TLX Ultracentrifuge at 4 °C, and centrifuged at 200,000*g* for 45 min in series to remove other nonmembrane subcellular fractions. The resulting cell membrane pellet was stored at −20 °C until further processing. Optimizations to this approach have been shown to generate a purified cell membrane pellet (28).

### Enzymatic Release and Purification of N-glycans

Proteins were suspended with 100 μl of 100 mM NH_4_HCO_3_ in 5 mM DTT and heated at 100 °C for 10 s to thermally denature the proteins. To release the glycans, 2 μl of peptide N-glycosidase F were added to the samples, followed by incubation in a microwave reaction at 60 °C for 10 min to accelerate N-glycan release. Samples were incubated for 18 h at 37 °C to hydrolyze the N-glycans. The reaction was quenched with 350 μl of water followed by ultracentrifugation at 200,000*g* to separate the N-glycans and the MF containing our lipids and deglycosylated proteins. The released N-glycans were purified by solid-phase extraction using porous graphitized carbon–packed cartridges (GlyGen; catalog no.: FNSCAR800). The cartridges were first equilibrated with nanopure water and a solution of 80% (v/v) acetonitrile and 0.05% (v/v) trifluoroacetic acid in water. The dried samples were solubilized, loaded onto the cartridge, and washed with nanopure water to remove salts and buffer. N-glycans were eluted with a solution of 40% (v/v) acetonitrile and 0.05% (v/v) trifluoroacetic acid in water, dried, and reconstituted in 30 μl of water prior to mass spectrometric analysis.

### Glycoproteomics Enzymatic Digestion and Purification

Brain regions of interest were homogenized, and cell lysis was performed in a buffer containing 0.25 M sucrose, 20 mM Hepes–KOH (pH 7.4) using a sonicator with standard probe. Subcellular fractionation was performed to isolate cell MF. Cell membrane proteins were dissolved and denatured with 8 M urea for optimal digestion followed by DTT and alkylated with iodoacetamide. Protein concentration was determined using Bicinchoninic acid Protein Assay Kit. Samples were then digested with trypsin (sequencing grade modified; Promega; catalog no.: V5111) at 37 °C for 18 h. Enrichment of glycopeptides was necessary to avoid ion suppression effects from coeluting peptides. The high enrichment efficiency was obtained using iSPE hydrophilic interaction LC cartridges (Hilicon; catalog no.: 200.001.0100).

### Glycomics Analysis by LC–MS/MS

Purified brain N-glycans were analyzed using an Agilent nano-LC/chip Q-ToF MS system. The nano-LC system employs a binary solvent consisting of A (0.1% formic acid in 3% acetonitrile in water [v/v]) and B (0.1% formic acid in 90% acetonitrile in water [v/v]). Samples were enriched and separated on the Agilent HPLC-Chip comprised of a 40 nl enrichment column and a 75 μm × 43 mm ID analytical column both packed with porous graphitized carbon in 5 μm particle size. The sample was delivered by the capillary pump to the enrichment column at a flow rate of 3 μl min^−1^ and separated on the analytical column by the nanopump at a flow rate of 0.3 μl min^−1^ with a gradient that was previously optimized for N-glycans: 0% B, 0 to 2.5 min; 0 to 16% B, 2.5 to 20 min; 16 to 44% B, 20 to 30 min; 44 to 100% B, 30 to 35 min; and 100% B, 35 to 45 min followed by pure A for 20 min of equilibration. MS spectra were acquired at 1.5 s per spectrum over a mass range of *m/z* 600 to 2000 in positive ionization mode. Mass inaccuracies were corrected with reference mass *m/z* of 1221.991.

N-glycan compositions were identified using MS and MS/MS data as well as an in-house retrosynthetic library based on the mammalian N-glycan biosynthetic pathway. Deconvoluted masses were compared with theoretical masses using a mass tolerance of 20 ppm and a false discovery rate of 0.5% on the Agilent MassHunter software, version B.7. Relative abundances were determined by integrating peak areas for observed glycan masses, averaging abundances from instrumental triplicates and normalizing to the summed peak areas of all glycans detected.

Enriched glycopeptides were subjected to nanoLC-Orbitrap Fusion Lumos for MS/MS analysis. One microliter of samples was injected, and the analytes were separated on an Acclaim PepMap C18 LC column (3 μm, 0.075 mm × 500 mm; Thermo Fisher Scientific) at a flow rate of 300 nl min^−1^. Binary mobile phase containing 0.1% formic acid in water and 80% acetonitrile containing 0.1% formic acid were used as solvents A and B, respectively. MS spectra were acquired at a rate of 1.5 s per spectrum over a mass range of *m/z* 600 to 2000 in positive ionization mode. The filtered precursor ions in each MS spectrum were subjected to fragmentation through 30% higher-energy C-trap dissociation with nitrogen gas.

### Data Analysis

Glycoproteins were identified using the Byonic, version 3.5.0 software (Protein Metrics) against the *Homo sapiens* (human) protein database from UniProt, which corresponds to a protein count of 79,740 (protein ID: UP000005640). A precursor mass tolerance of 10 p.p.m. and fragmentation mass tolerance of 20 p.p.m. was used. The enzyme digestion parameters used included C-terminal cleavage by trypsin on cleavage site K and R with a maximum of two missed cleavages. Alkylation of cysteine with carbamidomethylation was assigned as a fixed modification. Deamidation of asparagine and glutamine and oxidation of methionine were selected as common variable modifications. Acetylation of the protein N terminus and methylation of lysine and arginine were assigned as rare variable modifications. An in-house human N-glycan database was applied for site specific in asparagine N-glycosylation. A DeltaMod threshold of 10.0 was used, and a |Log Prob| <2 (error probabilities >0.01) was removed.

Glycopeptides were quantified using Byologic, version 3.11-1 (Protein Metrics) after identification using Byonic. Glycopeptide intensities were normalized to each specific protein glycosite, allowing for comparison between glycoproteins having varying degrees of expression across brain regions. Glycans attached to each glycosite were categorized based on the N-glycan subtypes—high mannose, undecorated, fucosylated, sialylated, and sialofucosylated N-glycan types. Relative abundances of each N-glycan subtype attached to protein glycosites were summed and summarized into heat maps generated by GraphPad Prism, version 9 (GraphPad Sogftware, Inc). The heat maps were further annotated by mapping the gene ontologies—biological processes of the glycoproteins in PantherDB (http://pantherdb.org/) (30). This allows us to view the N-glycosylation of proteins participating in specific biological processes for each brain region.

## Results

### Human Brain Global N-Glycome Profiling in a Region-Specific Manner

**Fig. 1:**
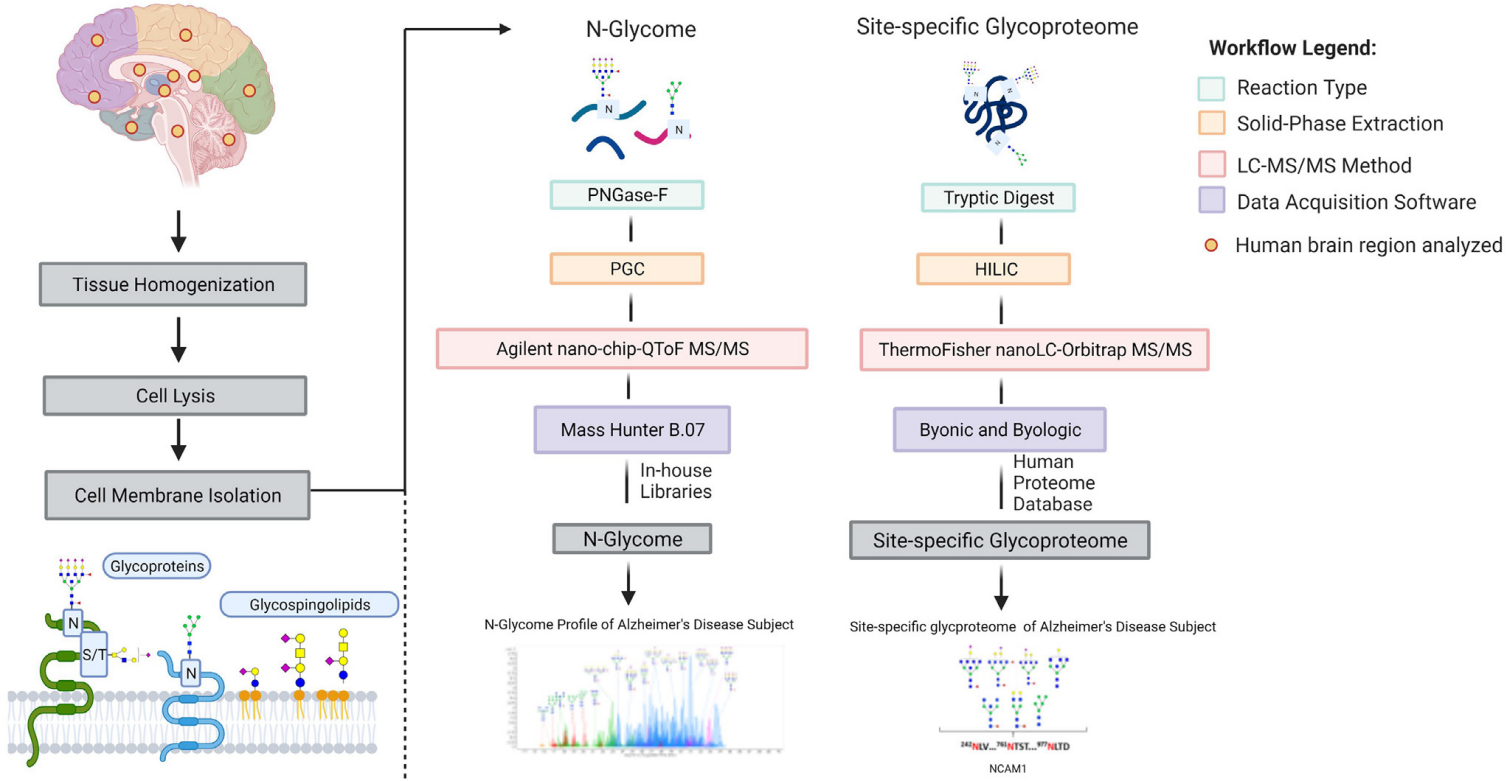
**Summary of the workflow for comprehensive LC–MS/MS analysis of the human brain.** Tissue region of interest was subjected to tissue homogenization followed by cell lysis to obtain cell membrane fraction. Two cell membrane fractions were isolated from the same brain region, one for N-glycomic analysis and the other for site-specific glycoproteomic analysis. The reaction type, solid phase extraction, LC–MS method, and data-acquisition software are shown.

**Fig. 2:**
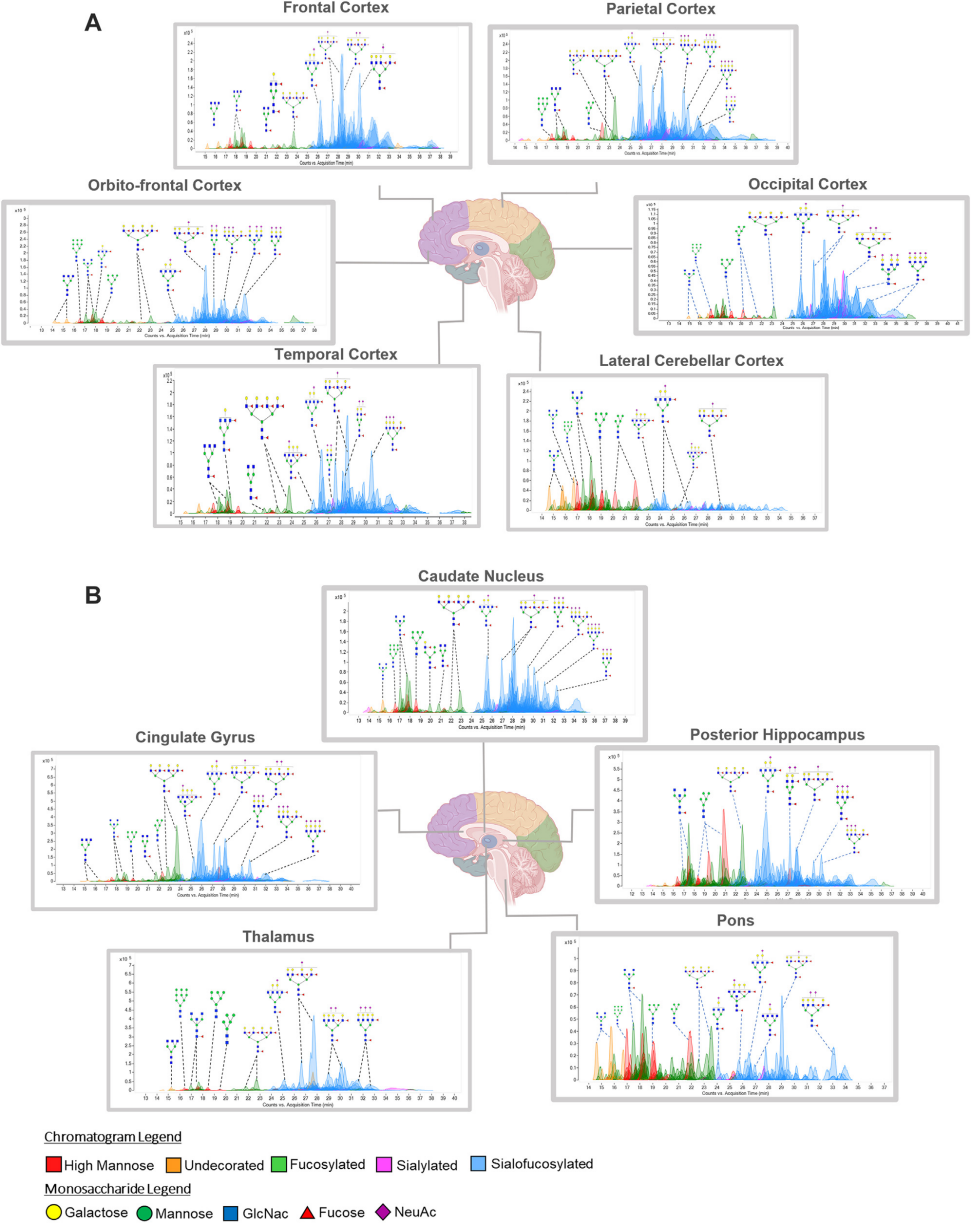
**N-glycome map of the 11 functional brain regions for NCI-72.** For (*A*) outer regions and (*B*) inner regions. Representative total compound chromatograms of N-glycans from the temporal cortex, orbitofrontal cortex, frontal cortex, parietal cortex, occipital cortex, lateral cerebellar cortex, thalamus, cingulate gyrus, caudate nucleus posterior hippocampus, and pons obtained by PGC-chip-QToF MS/MS. Representative putative structures were assigned to each peak based on monosaccharide compositions. NCI, no cognitive impairment.

Brain sample preparation involved tissue homogenization, a series of ultracentrifugation for cell membrane enrichment, and glycan release with the enzyme peptide N-glycosidase F (Fig. 1). A separate sample set was similarly prepared without the N-glycan release for glycoproteomic analyses. Representative nanoLC–MS chromatograms of glycans associated with the different regions for the 72-year-old NCI subject (NCI-72) are presented in Figure 2. The outer brain regions were grouped in the upper presentation (Fig. 2*A*), whereas the inner regions were in the lower presentation (Fig. 2*B*). In each chromatogram, over 200 compositions were observed across four orders of magnitude in dynamic range. The figures were color coded according to their structural features with the most common glycoform corresponding to sialofucosylated species (Fig. 2, *blue*). The other glycoforms included undecorated (*orange*), high mannose type (*red*), sialylated only (*pink*), and fucosylated only (*green*). Non–high mannose–type glycans may be either hybrid or complex types; however, the latter are far more abundant. Based on the chromatogram, spatial (or regional) variations were readily observed even with casual visual inspections. Closer inspection of the NCI-72 brain N-glycome reveals that sialofucosylated species account for more than 70% of the entire N-glycome within eight of the brain regions including the frontal, temporal, parietal, occipital, cingulate, and orbitofrontal cortex, caudate nucleus, and thalamus. The remaining three brain regions, which include the posterior hippocampus, lateral cerebellar cortex, and pons, displayed lower levels of sialofucosylated compositions with relative abundances of 55, 29, and 28%, respectively (supplemental Fig. S1, *A*–*K*). Fucosylated-only type N-glycans were the second most abundant glycoform in NCI-72. The highest levels of fucosylated-only N-glycans ranged from 29% to 38% and were found in the posterior hippocampus, lateral cerebellar cortex, and pons. The remaining eight brain regions had lower levels of fucosylated-only species ranging from 5% to 12%. The lateral cerebellar cortex and pons contained 23% of the high-mannose structures, which are the highest levels compared with the other regions of NCI-72.

**Fig. 3:**
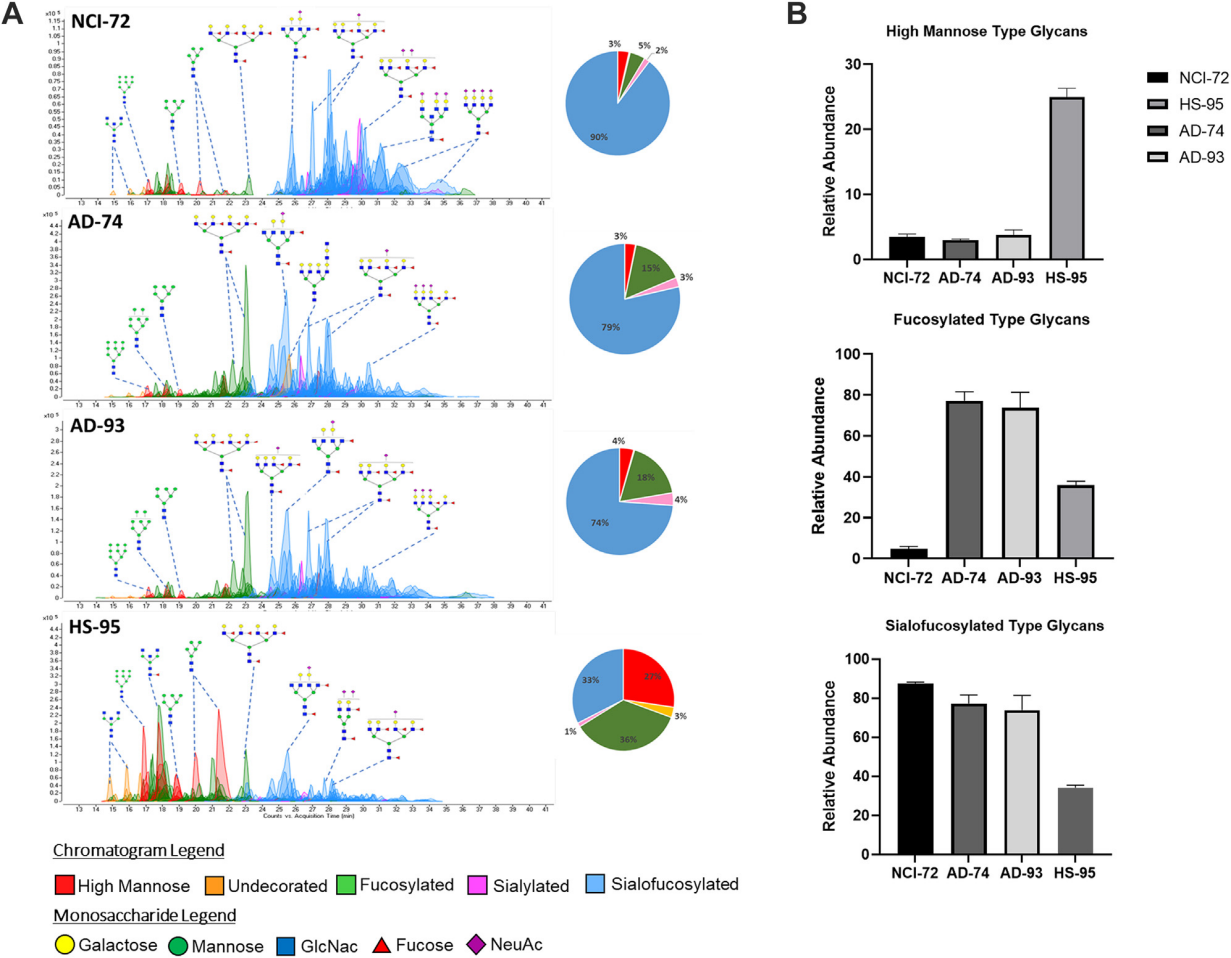
**Comparison of N-glycomic profiles of the 4 subjects in a single region.***A*, representative total compound chromatograms of N-glycome from the occipital cortex from subjects NCI-72 (*top*), AD-74, AD-93, and HS-95 (*bottom*). Monosaccharide schematic symbols: *yellow circle*, galactose; *green circle*, mannose; *blue square*, N-acetyl glucosamine; *red triangle*, fucose; and *purple diamond*, N-acetylneuraminic acid. High mannose glycans correspond to peaks in *red*. N-glycans without fucose or sialic acid termed undecorated correspond to peaks in *orange*. Peaks in *green* correspond to N-glycans that are fucosylated only with no sialic acid. Peaks in *blue* corresponds to N-glycans with both fucose and sialic acid moiety termed sialofucosylated. *B*, bar graphs representing abundances of high mannose, fucosylated- and sialofucosylated-type N-glycans. Relative abundance of subjects NCI-72, AD-74, AD-93, and HS-95 from the occipital cortex brain region. Error bars indicate instrumental replicates. AD, Alzheimer’s disease; HS, hippocampal sclerosis; NCI, no cognitive impairment.

Variations between the four subjects were examined in the different regions. LC–MS chromatograms of the occipital cortex, a brain region important in vision and visual interpretation, showed wide variations in N-glycan compositions among the four subjects NCI-72, AD-74, AD-93, and HS-95 (Fig. 3*A*). Variations were observed in the two non-AD subjects (NCI-72 and HS-95), which had widely different types of glycans, with NCI-72 having very little high mannose abundances and primarily complex-type structures that were sialofucosylated. HS-95 had very high levels of high mannose-type glycans and low levels of complex-type sialofucosylated structures. The N-glycome of HS-95 showed a more varied distribution of high mannose (27%), fucosylated-only (36%), and sialofucosylated (33%) species (Fig. 3*B*). Interestingly, the two AD subjects (AD-74 and AD-93) yielded highly similar chromatograms. For example, the N-glycan composition Hex_3_HexNAc_5_Fuc_1_NeuAc_0_ has similar abundances in AD-74 and AD-93 but was sixfold higher between NCI-72 and HS-95. In addition, large variations were also observed in the frontal cortex, a region primarily responsible for controlling executive functions including working memory and cognitive flexibility (31). Comparison of AD-74 and NCI-72 showed that sialofucosylated N-glycans were of lesser abundance in AD-74 (supplemental Fig. S1). Interestingly, the sialofucosylated glycans that decreased contained high degrees of terminal sialylation represented by compositions such as Hex_7_HexNAc_6_Fuc_2_NeuAc_4_, Hex_7_HexNAc_6_Fuc_3_NeuAc_3_, Hex_7_HexNAc_6_Fuc_4_NeuAc_2_, and Hex_7_HexNAc_6_Fuc_3_NeuAc_3_. A similar comparison of the other regions is presented in supplemental Fig. S1 with more in-depth analyses provided later.

### N-Glycomic Variations in the Brain Regions

**Fig. 4:**
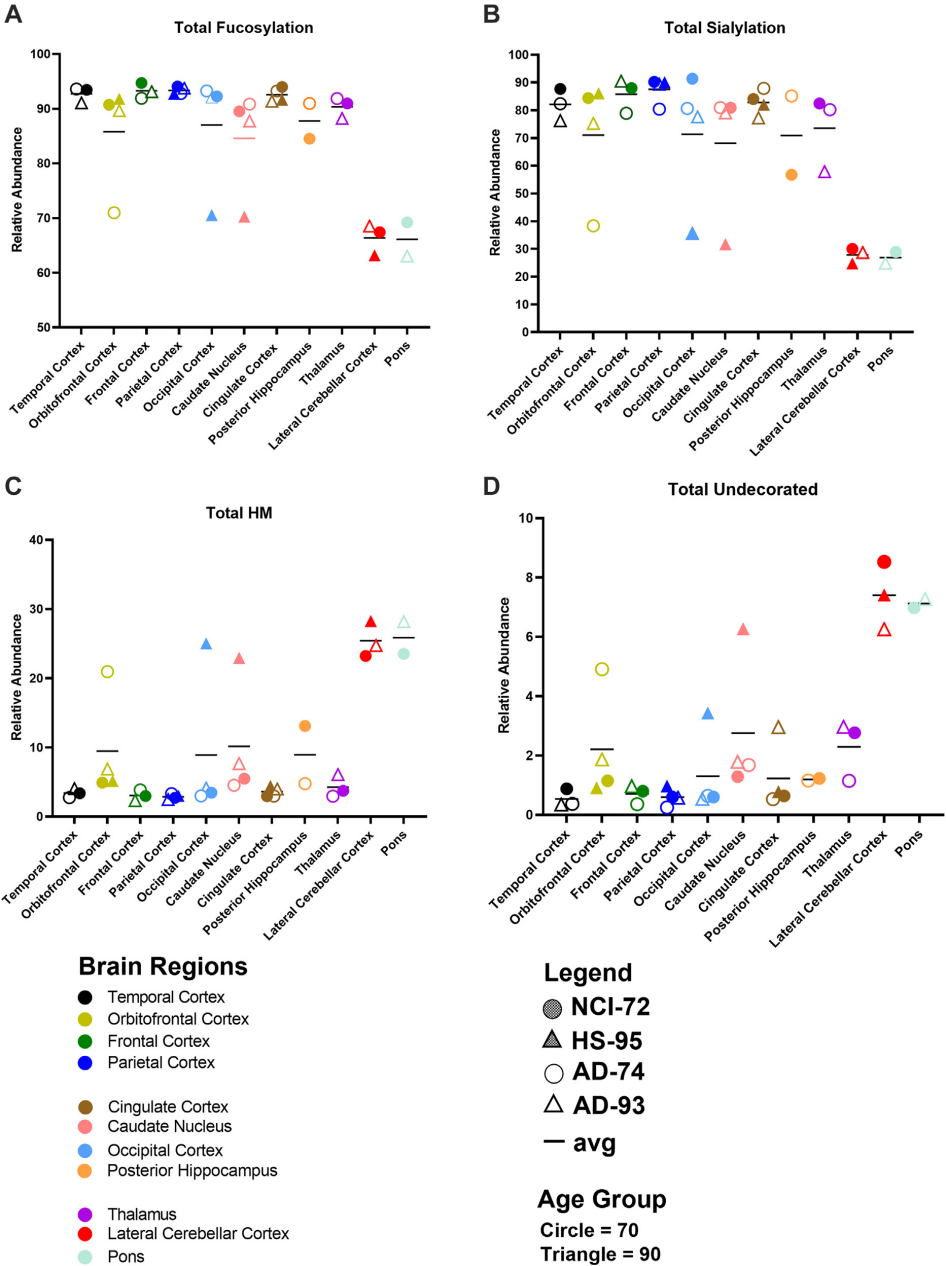
**The relative abundances of the total amounts of fucosylation, sialylation, high mannose, and undecorated.** (*A*) Fucosylation, (B) sialylation, (*C*) high mannose, and (*D*) undecorated across the 11 brain regions from the four subjects. Each brain region is distinctly color coded where *circles* denote age group 70 and *triangles* denote age group 90. *Shaded shapes* represent subject with no cognitive impairment (NCI), whereas unshaded shapes represent subject with hippocampal sclerosis (HS).

N-glycans detected in the individual brain regions ranged in numbers between 200 and 900 (including isomers based on chromatographic separation), revealing the large number of N-glycan compositions across the brain (supplemental Fig. S2). All glycan species were grouped into the five subgroups that included undecorated (complex and hybrid types lacking in fucose or sialic acid), high mannose, fucosylated-only, sialylated-only, and sialofucosylated (glycans containing both sialic acid and fucose moieties) (Figs. 2 and S1*A*). To monitor the total levels of fucosylation, both fucosylated-only and sialofucosylated types were grouped (Fig. 4). Similarly, total levels of sialylation involved grouping sialylated-only and sialofucosylated species. Brain N-glycans were generally high in fucose and sialic acids, and in nearly all regions, fucosylated N-glycans represented over 90% of the total abundances (Fig. 4*A*). Similarly, sialylated glycans represented 70 to 90% of all brain N-glycans (Fig. 4*B*). As both sialylation and fucosylation were abundant, most of the N-glycans in nearly all the regions were sialofucosylated. The exceptions were the lateral cerebral cortex and pons where sialofucosylation was lower in comparison to the other nine brain regions but still high at 60 to 70% (relative abundances). The lower abundant N-glycans were high mannose and undecorated (Fig. 4, *C* and *D*). High mannose glycans were generally low in abundances (below 10%) except in the lateral cerebral cortex and pons, where they represent 25% of the total glycan species. Those that were non–high mannose–type glycans were composed primarily of over 65% complex types with lower abundances (around 15%) for hybrid-type structures (supplemental Fig. S3).

**Fig. 5:**
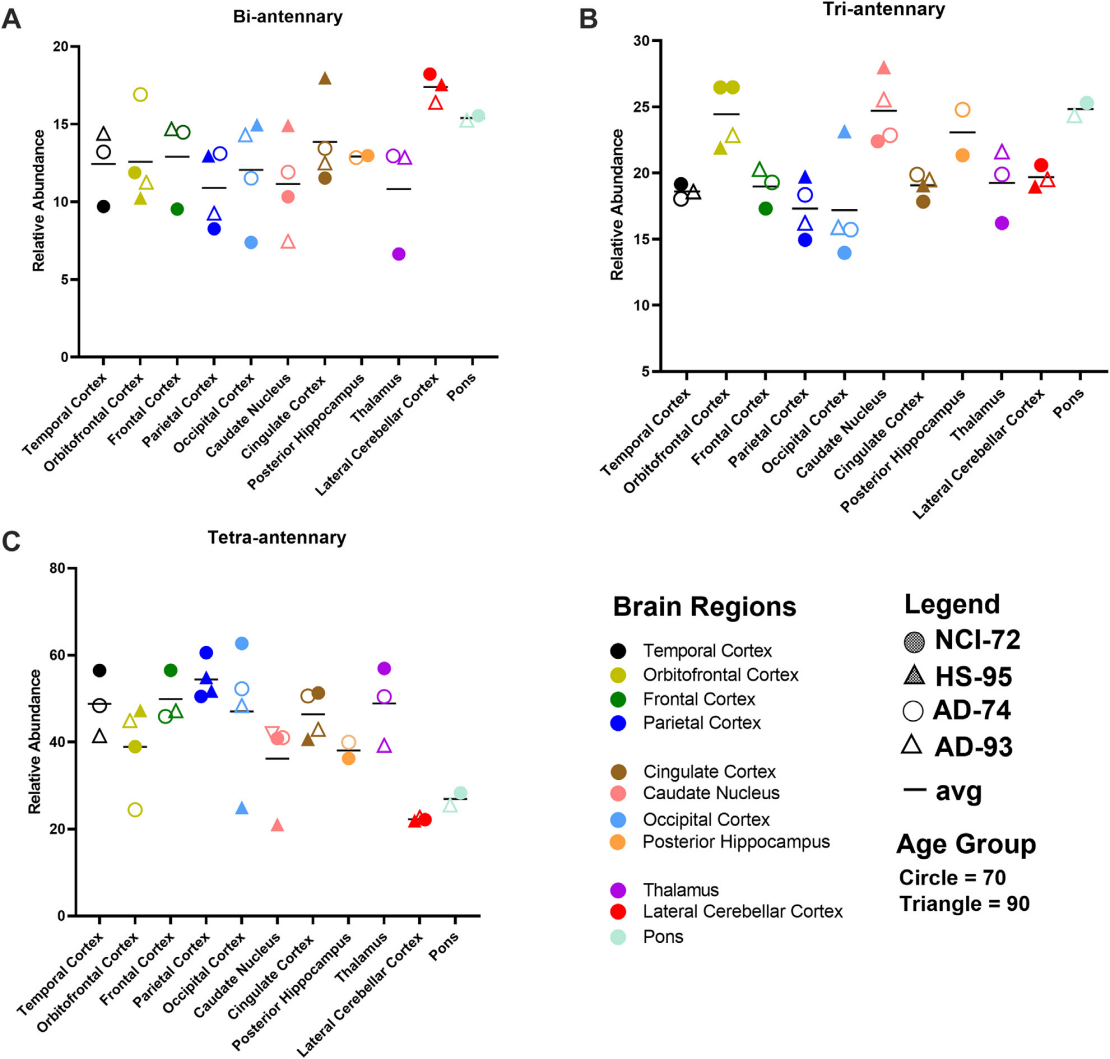
**Comparison of the level of branching**. For (*A*) biantennary, (*B*) triantennary, and (*C*) tetra-antennary across the temporal cortex, orbitofrontal cortex, frontal cortex, parietal cortex, cingulate cortex, caudate nucleus, occipital cortex, posterior hippocampus, thalamus, lateral cerebellar cortex, and pons.

We also evaluated the level of branching in the N-glycans across all the brain regions (Fig. 5). The N-glycans were generally highly branched with tetra-antennary type glycans being the most abundant with relative intensities ranging from 22% to 60% (Fig. 5*C*). However, in the lateral cerebellar cortex and pons, similar abundances of biantennary, triantennary, and tetra-antennary structures were observed. The highest abundances of tetra-antennary glycans were in the parietal cortex for all four subjects. Biantennary glycans were found to be highest in the lateral cerebellar cortex with almost 20% in relative abundances (Fig. 5*A*). Triantennary glycans ranged from 16% to 24% with the highest percentages observed in the orbitofrontal cortex and caudate nucleus (Fig. 5*B*).

A comprehensive representation of all detected N-glycans in the brain regions was summarized (supplemental Fig. S4). The results indeed showed that sialofucosylated N-glycans were the most abundant in almost all brain regions. The exceptions were again noted in the occipital cortex and caudate nucleus of NC-95 and the orbitofrontal of AD-74. For all the subjects, the lateral cerebellar cortex and pons differed from the rest of the regions. It should be noted that there were specific N-glycans that were abundant in the samples, regardless of age, disease, and brain region. Notably, the sialofucosylated tetra-antennary species with composition Man_3_Gal_3_GlcNAc_7_Fuc_4_NeuAc was abundant across 78% of all the samples (supplemental Fig. S4).

A nontargeted analysis of the four subjects using principal component analysis and all detected N-glycan compositions with their relative abundances was performed (supplemental Fig. S5). The results showed brain regions clustered into three distinct groups (supplemental Fig. S5). Samples from NCI-72 clustered in three different groups. One group (*cluster 1*) was composed of the orbital frontal cortex, caudate nucleus, temporal cortex, frontal cortex, thalamus, occipital cortex, parietal cortex, and cingulate cortex. The posterior hippocampus of NCI-72 was grouped into *cluster 2*. The remainder, lateral cerebellar cortex, and pons, of NCI-72 associated with a third group (*cluster 3*). For the 95 year old with HS (HS-95), *cluster 1* also contained the orbital frontal cortex, temporal cortex, frontal cortex, and parietal cortex; however, the caudate nucleus and occipital cortex grouped with *cluster 3*, whereas the cingulate cortex grouped with *cluster 2*. Because of difficulties in obtaining all the brain regions, some such as the thalamus were not obtained for all subjects. The lateral cerebellar cortex, which was previously in *cluster 3*, remained unchanged. AD-74 showed large differences compared with NCI-72. The caudate nucleus, thalamus, and cingulate cortex remained in *cluster 1*, whereas the temporal cortex, occipital cortex, frontal cortex, and parietal cortex, which were previously grouped in *cluster 1* and are the four major sections of the outer brain lobes, now grouped with *cluster 2*. *Cluster 3* only included the orbitofrontal cortex for AD-74. Finally, for the 94 year old with AD (AD-94), *cluster 1* was composed of the orbitofrontal cortex, caudate nucleus, and frontal cortex. *Cluster 2* consisted of the cingulate cortex, temporal cortex, occipital cortex, and thalamus. Interestingly, *cluster 3*, which included the pons and lateral cerebellar cortex for all subjects, showed the strongest glycan diversity containing high levels of high mannose–type species. Changes in glycosylation were apparent in some brain regions. Namely, the caudate nucleus of HS-95 was found in *cluster 3*, whereas for the other subjects, this brain region grouped in *cluster 1*. Similarly, for the occipital cortex, HS-95 grouped in *cluster 3*, whereas AD-74 and AD-95 grouped in *cluster 2*, and NCI-72 grouped in *cluster 1*.

### Glycoproteomic Analysis of the Brain Regions

A glycoproteomic analysis of the membrane proteins in the select brain regions (frontal, temporal, parietal, occipital, cingulate, lateral cerebellar and orbitofrontal cortex, posterior hippocampus, thalamus, caudate nucleus, and pons) was also performed. Because the glycans were primarily on the membrane, membrane proteins were enriched and analyzed using standard proteomic analysis. This yielded approximately 1000 proteins in each of the regions. An additional digested MF was passed through hydrophilic interaction LC solid phase extraction to enrich for glycopeptides. The region-specific cell membrane glycoproteins were identified utilizing the targeted N-glycome generated from each brain region, and site-specific glycosylation was determined and quantified. The number of site-specific glycopeptides varied with each region, accounting for a total of more than 500 unique membrane glycoproteins identified when all regions were combined.

The glycoproteins in each region were compared across all 11 regions. All glycoproteins detected in a specific region for the four subjects were grouped together (supplemental Fig. S6). Comparisons yielded similarities in the membrane glycoproteins between the regions. Comparing the temporal cortex and the frontal cortex yielded a 47% similarity. The similarities between the regions differed considerably from as low as 9% between the parietal cortex and pons to as high as 60% for the orbitofrontal cortex and caudate nucleus. Interestingly, the geographic location does not seem to correlate with glycoprotein similarities. For example, the parietal cortex that sits near the top and center of the cerebral cortex has low glycoprotein similarities with the pons, which is not part of the cerebral cortex but the brainstem. Remarkably, a very high overlap in membrane proteins is observed in the orbitofrontal cortex, which sits in the front of the cerebral cortex, and the caudate nucleus, which lies deep inside the brain.

**Fig. 6:**
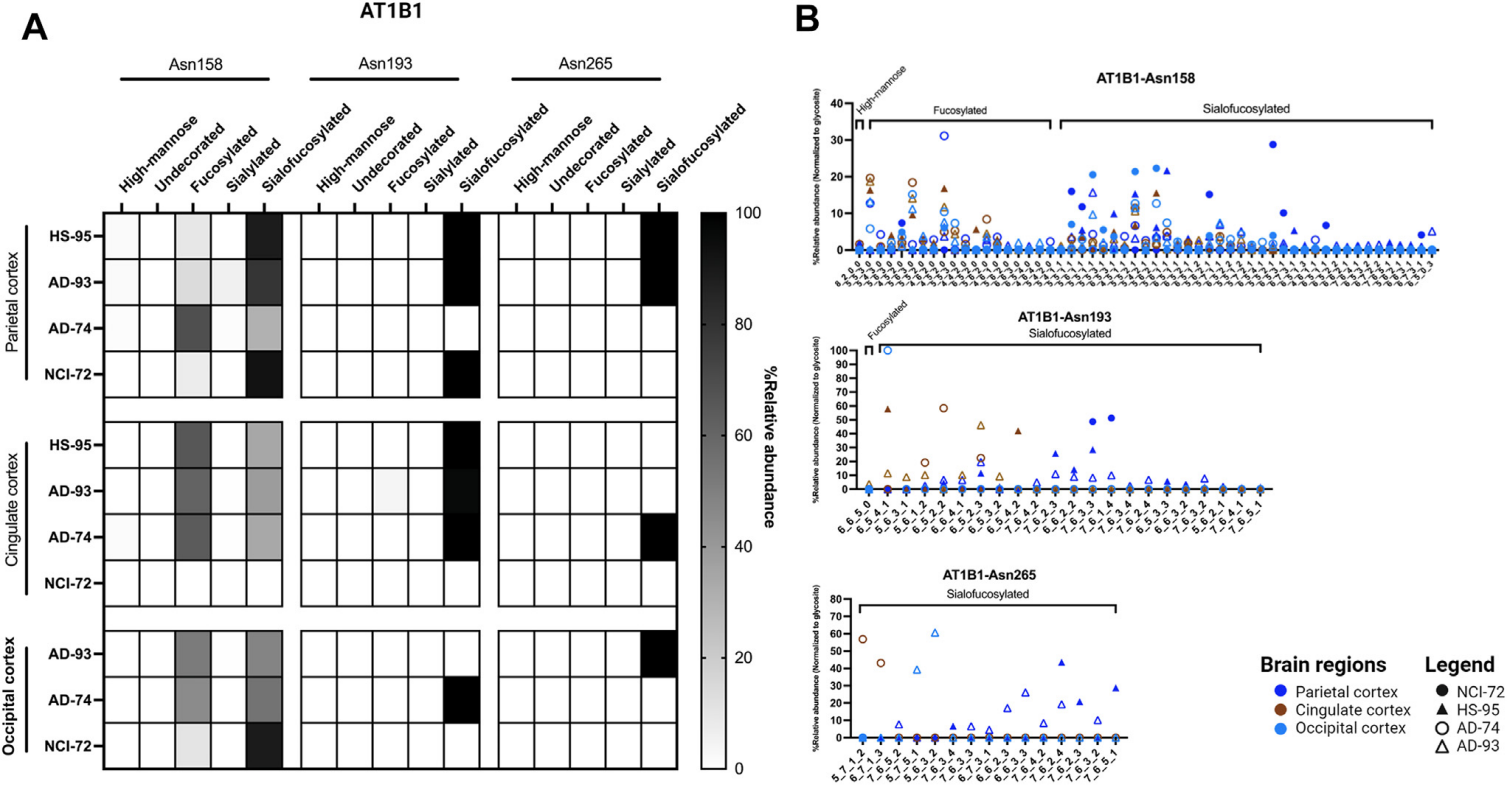
**Changes in glycosylation of protein AT1B1 across brain regions.***A*, site-specific glycosylation pattern of AT1B1 (sodium/potassium-transporting ATPase subunit B1) across three brain regions—parietal, cingulate, and occipital cortex—and across its three known glycosylation sites: Asn158, Asn193, and Asn265. *B*, distribution of glycoforms and glycan type across the three brain regions and three glycosylation sites.

Glycoproteins varied in glycosylation between different glycosites. The proteins sodium/potassium-transporting ATPase subunits beta-1 (AT1B1), beta-2 (AT1B2), and beta-3 (AT1B3) were found to be abundant in all regions and illustrated the diversity between glycosylation sites and brain regions. AT1B1 had three putative N-glycosites corresponding to N158, N193, and N265. AT1B1-N158 was found to be both fucosylated and sialofucosylated, while N193 and N265 were solely sialofucosylated (Fig. 6*A*). The most abundant N-glycan compositions in N158 were found to be sialofucosylated (mainly monosialylated) with compositions corresponding to Hex_5_HexNAc_5_Fuc_1_NeuAc_1_, Hex_5_HexNAc_5_Fuc_2_NeuAc_1_, and Hex_5_HexNAc_6_Fuc_2_NeuAc_1_. N193 and N265 bore similar compositions but were more highly sialylated with di-, tri-, and tetra-sialylated to include Hex_6_HexNAc_5_Fuc_2_NeuAc_3_, Hex_7_HexNAc_6_Fuc_3_NeuAc_3_, and Hex_7_HexNAc_6_Fuc_2_NeuAc_4_ (Fig. 6*B*). For comparison, the relative abundance of each N-glycan composition was normalized to all the glycoforms associated with the site.

**Fig. 7:**
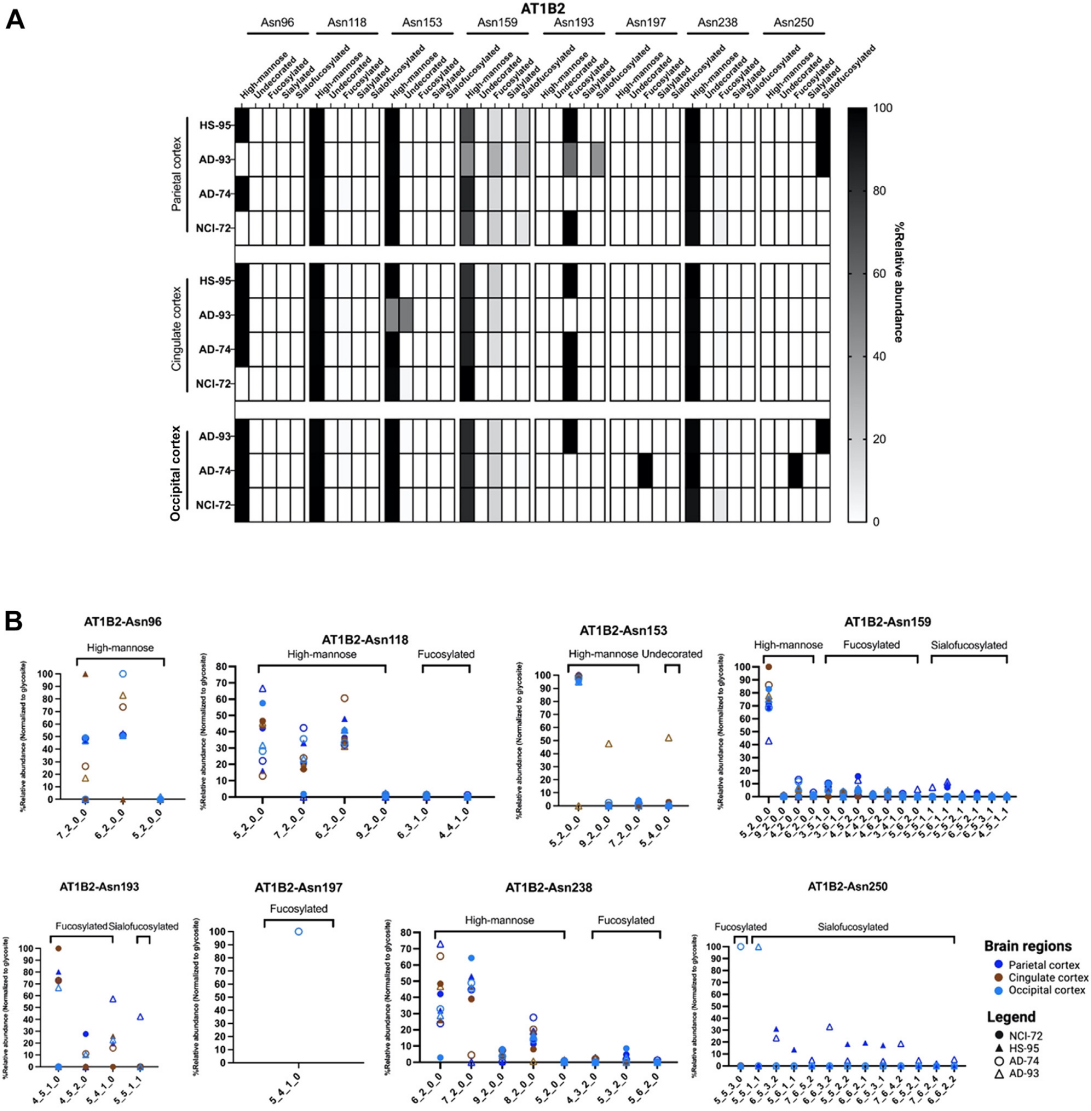
**Changes in glycosylation of protein AT1B2 across brain regions.***A*, site-specific glycosylation pattern of AT1B2 (sodium/potassium-transporting ATPase subunit B2) across three brain regions—parietal, cingulate, and occipital cortices—and across its eight known glycosylation sites: Asn96, Asn118, Asn153, Asn159, Asn193, Asn197, Asn238, and Asn250. *B*, distribution of glycoforms and glycan type across the three brain regions and eight glycosylation sites.

**Fig. 8:**
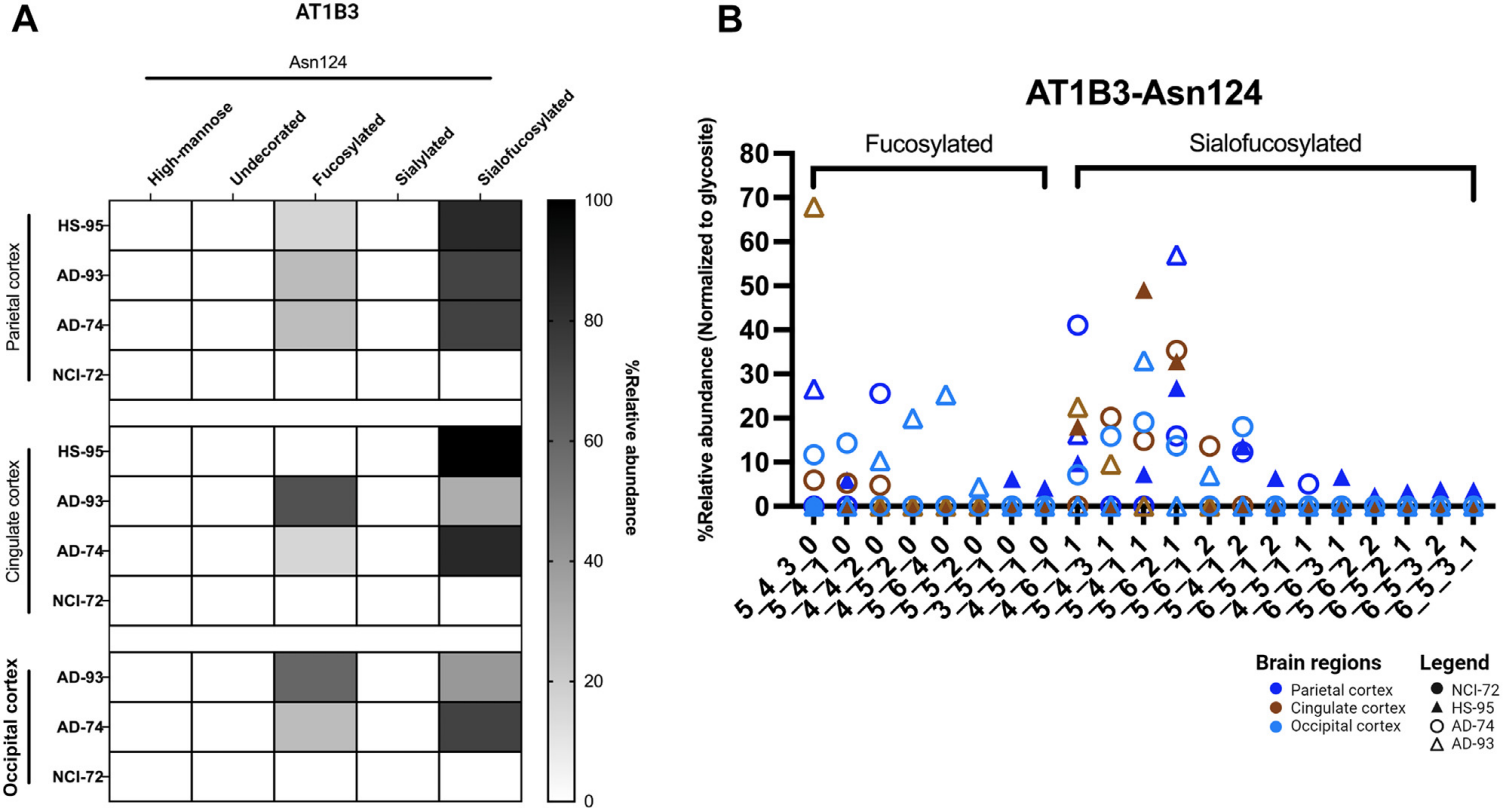
**Changes in glycosylation of protein AT1B3 across brain regions.***A*, site-specific glycosylation pattern of AT1B3 (sodium/potassium-transporting ATPase subunit B3) across three brain regions—parietal, cingulate, and occipital cortices—and across one of its known glycosylation sites: Asn124. *B*, distribution of glycoforms and glycan type across the three brain regions and glycosylation site.

To determine whether site-specific occupancy of proteins varied across regions, selected glycoproteins were compared across three randomly selected regions, namely the parietal-, occipital- and cingulate cortex. N-Glycans in the three proteins AT1B1, AT1B2, and AT1B3 were found to be primarily fucosylated and sialofucosylated complex–type glycans and high mannose types. For AT1B1, there were only minor changes in glycosylation between the three regions. However, AT1B2 with eighth detected glycosites, the glycoforms varied between regions and between subjects. The glycosites N96, N118, N153, N159, and N238 were primarily mannosylated, whereas N193 and N250 were mainly fucosylated and sialofucosylated (Fig. 7*A*). Interestingly, site N197 was only glycosylated in the occipital cortex of AD-74 and was not found to be glycosylated in the other two regions. In glycosite N159, Man5 was the most abundant glycoform; however, fucosylated and sialofucosylated composition were still present in lower abundances (Fig. 7*B*). AT1B3 was found to be least glycosylated among the three ATPase beta subunits, with only one N-glycosylation site, N124. In the three brain regions, AT1B3-N124 was found to be primarily sialofucosylated and fucosylated (Fig. 8*A*).

**Fig. 9:**
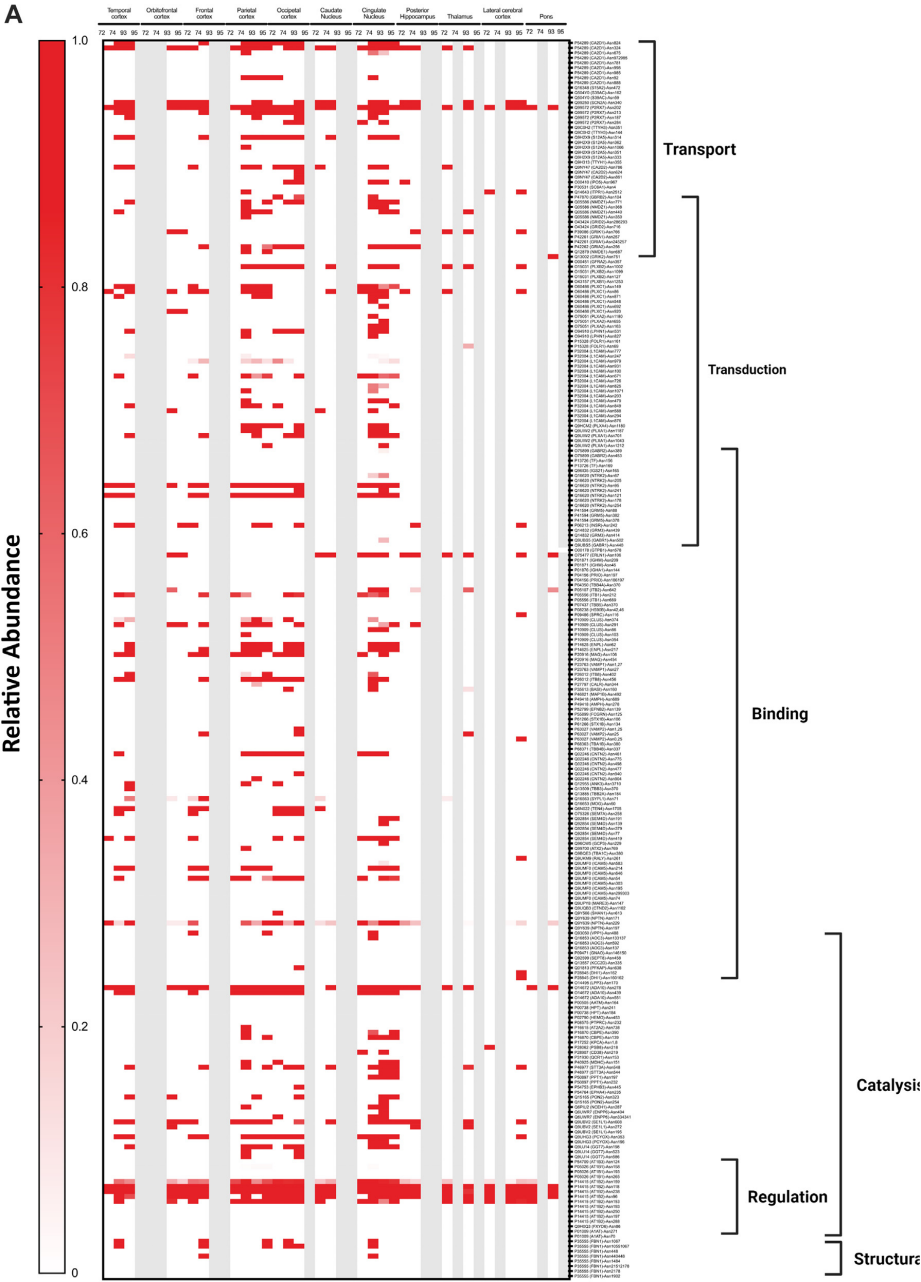

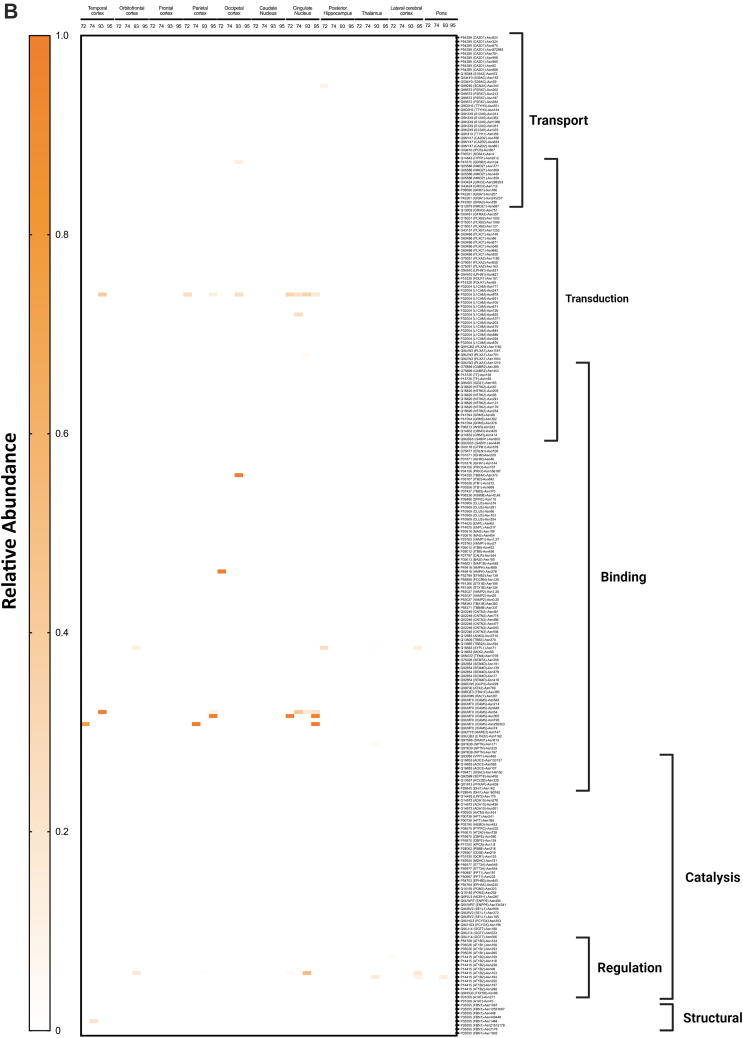

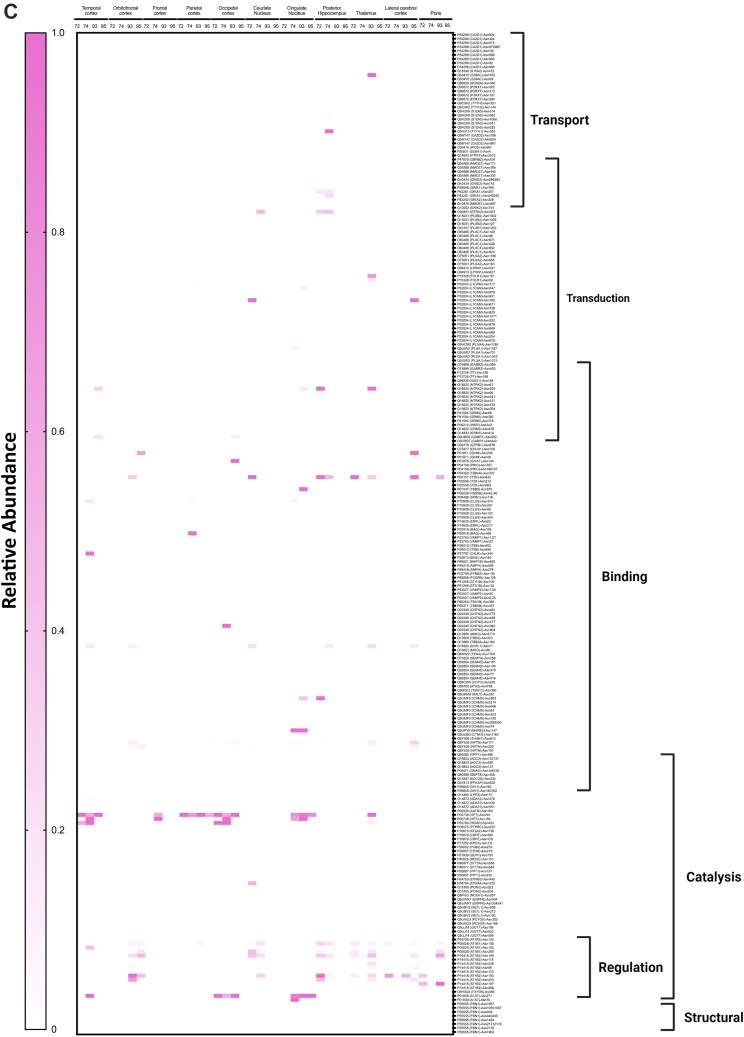

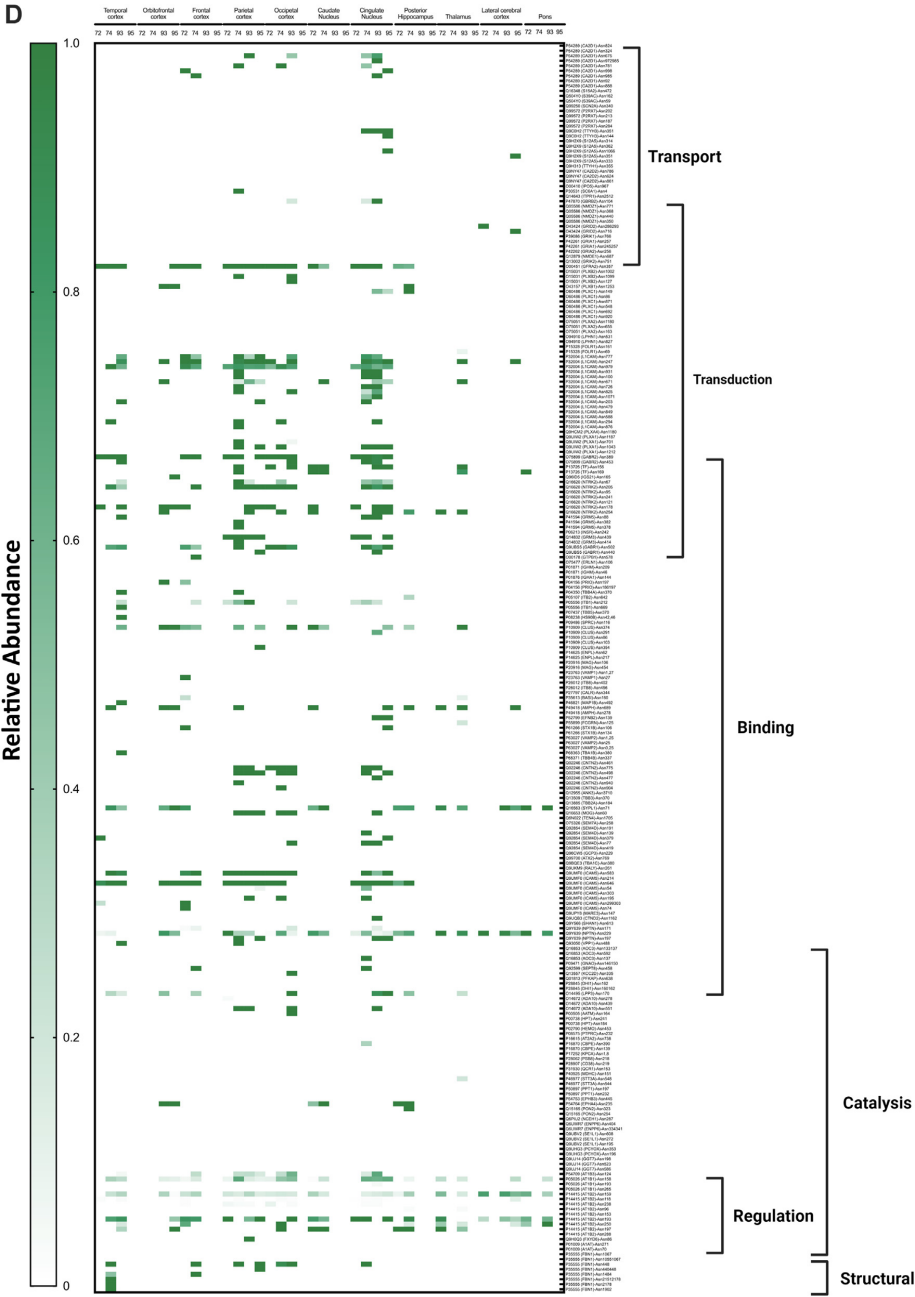

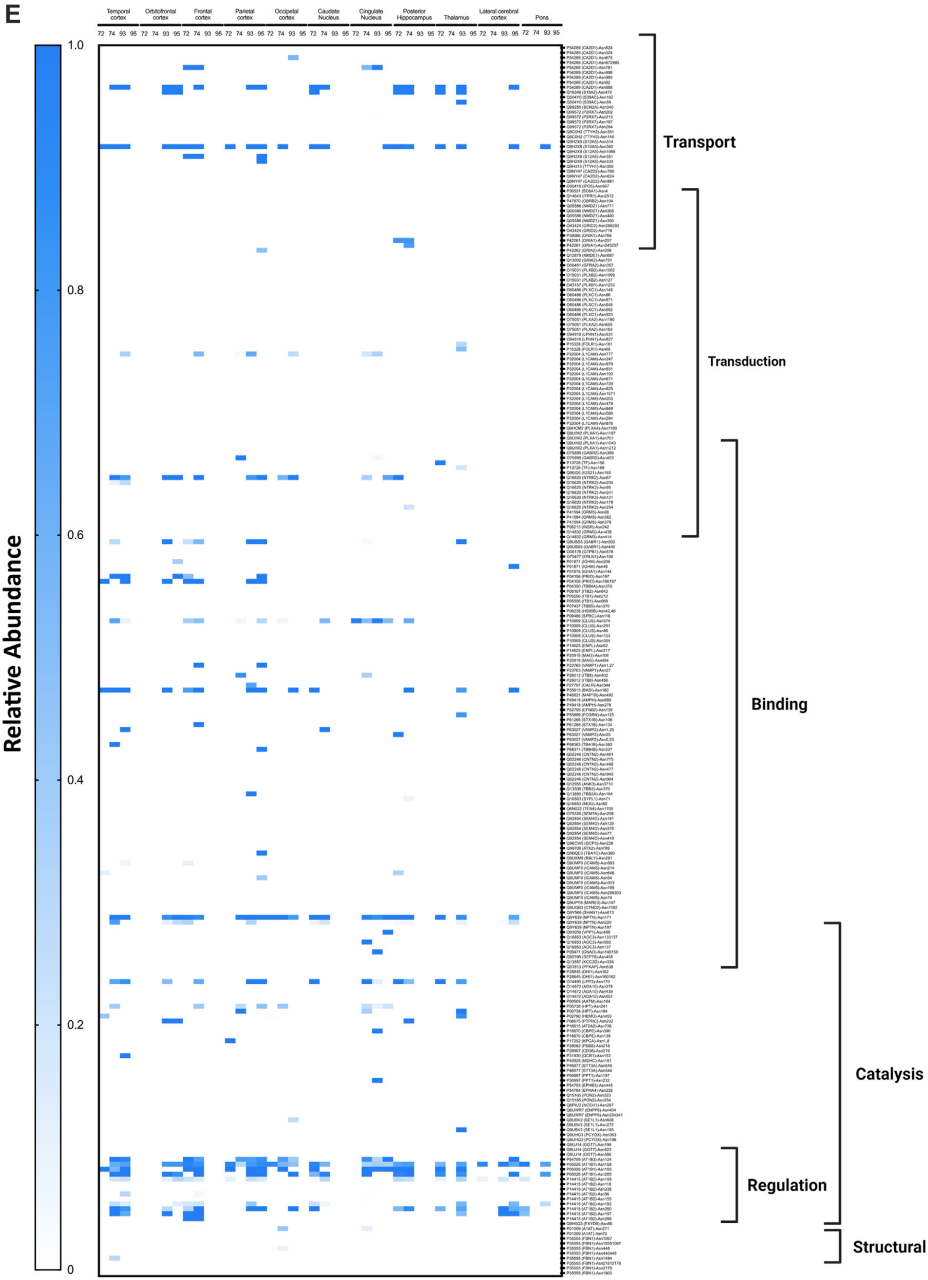
**Heat maps displaying the characterization of the N-glycoproteome in the human brain separated by their glycotypes.** Glycotypes including (*A*) high mannose, (*B*) undecorated, (*C*) sialylated-only, (*D*) fucosylated-only, and (*E*) sialofucosylated. The heat map displays low to high relative abundances. More intense color correlated with higher glycan abundances on the specific glycosite. Molecular processes that include transport, transduction, binding, structural, regulation, and catalysis are shown on the *right*.

To obtain a comprehensive view of the glycans associated with the glycoproteins in the different regions, heat maps were constructed corresponding to each glycosite across different regions (Fig. 9*A*). Each horizontal line represented a specific glycosite, with the glycosites for the same proteins grouped together. The proteins were further grouped according to their function as determined by the gene ontology. The results were separated for the different glycans with the high mannose-type glycans illustrated in Figure 9*A*, complex-type undecorated glycans (Fig. 9*B*), complex-type sialylated-only glycans (Fig. 9*C*), complex-type fucosylated-only glycans (Fig. 9*D*), and complex-type sialofucosylated glycans (Fig. 9*E*).

The high mannose–type glycans were found in most of the glycoproteins (Fig. 9*A*). Interestingly, the glycomic data showed that this glycans type was not particularly abundant in the brain. However, they appear to be widely distributed on several protein sites with some maintained throughout all subjects and brain regions (*solid red lines*). Glycoprotein AT1B2 glycosite N96, N118, and N238, a disintegrin and metalloproteinase domain–containing protein 10 (ADA10) at N278 and P2X purinoceptor 7 (P2RX7) at N202 followed similar behavior (Fig. 9*A*). The least abundant subtype in the glycomic profile was the undecorated N-glycans (Fig. 4*C*), which correlated well with the glycoproteomic analysis (Fig. 9*B*). There were no glycosites observed that maintained this glycan across all regions and subjects. However, intercellular adhesion molecule 5 at glycosite N303 was identified to contain the highest abundance of undecorated glycans in the cingulate cortex in subjects NCI-72 and HS-95 as well as temporal and parietal cortex in subject AD-93. Similarly, the sialylated-only glycans (Fig. 9*C*) were not broadly present but found in several more sites. However, these glycan subtypes were generally not found in the same glycosites among all regions and subjects. The most abundant sialylated-only glycopeptide was found on N241 of glycoprotein haptoglobin, which was present in more than 50% of the brain regions. Interestingly, the fucosylated-only (Fig. 9*D*) glycans were found in more sites than the undecorated and sialylated-only glycans. This glycan type was maintained among several regions and found on the majority of the glycoproteins. The most common complex type structures were the sialofucosylated species (Fig. 9*E*). These were similarly found in many glycosylation sites, some with the presence across several regions and all subjects.

## Discussion

The N-glycans are typically the most abundant of the glycoconjugates with the largest structural diversity. They provide a convenient yet extensive view of the brain glycome. Similarly, as many brain glycans are N-glycosylated, the geographical survey provides both glycomic and glycoproteomic distribution of the brain. Large glycomic variations were readily observed in different functional parts of the brain. The most notable observation in the N-glycome of the brain was the high abundance of sialofucosylated N-glycans as well as the presence of large multiantennary structures. Previous reports noted similarly multiantennary structures for brain glycans with as high as four and five antennae (21). These results contrast with N-glycans in the blood, the most glycomically characterized tissue, which yielded primarily biantennary structures with more sialylated but fewer sialofucosylated species (32). These large multiantennary structures and the high levels of sialofucosylation are appropriately given the highly interactive nature of brain cells and tissues (14). Studies performed both in rodents and pigs have also shown the primacy of sialic acid in improving cognitive functions (33, 34, 35). Fucose has been reported to play important roles in neural development (36, 37). Neuronal synapses have been shown to have enriched levels of fucose, and indeed increased levels of fucosyltransferases have also been reported during synaptogenesis (1, 38). Previous studies on brain glycosylation have focused primarily on sialylation (7, 33, 34, 39), but clearly, fucosylation is an abundant and therefore an important structural component. Indeed, high levels of fucosylation are found in most of the brain regions (Fig. 4*C*). When correlated with the glycoproteomic and gene ontology data, the fucosylated glycoproteins functionally align with molecular transduction (Fig. 9*D*) suggesting that high levels of fucosylated type glycan present in the brain are crucial in regulating many important cellular functions such as cell signaling. In addition, most of the fucose is in the core structures, which has been shown to promote trans protein–protein through the glycan rather than cis protein–protein interactions (40). There are two regions that are glycomically very dissimilar from the remaining brain regions and contain significantly lower sialofucosylated species. The lateral cerebellar cortex and pons are both low in complex-type structures and abundant in high mannose–type structures.

The variations between the subjects point to possible differences between disease states that could be revealed with more samples. For example, a comparison of the frontal cortex between NCI-72 and AD-74 (Figs. 4*C* and S1*B*) showed a decrease in sialofucosylated species. This result is in agreement with previous reports where a significant decrease in sialyltrasferase activity was similarly observed in serum, postmortem brains, and cerebrospinal fluid proteins of AD patients (41, 42, 43). Genomic studies have also associated the gene Siglec 33, a sialic acid–binding receptor, with late-onset AD (41). In these experiments, in the occipital cortex and temporal cortex, a decrease in sialofucosylated N-glycans was also observed when comparing NCI-72 with both AD subjects (AD-74 and AD-93). As the N-glycans were enriched from proteins on the cell membrane, the loss of these species, specifically the sialic acid moieties, may mean the loss of intracellular interactions on the cell surface of the brain cells. Studies involving neural cell adhesion molecules (NCAMs) showed that changes in glycosylation were associated with the disruption of several NCAM-dependent neurodevelopmental processes (44). A large body of work on brain sialylation has focused primarily on polysialic acid (34, 39, 45, 46); however, the large abundances of sialofucosylated species should encourage the examination of sialofucosylated NCAMs in axonal guidance.

The glycoproteomic analysis yielded site-specific information of proteins allowing direct comparison across brain regions and between subjects (Fig. 9). The glycans most preserved across sites are the high mannose types. These glycans are unique among the various types as some sites maintain them among all subjects and across many regions of the brain. Comparison of the other glycans shows that neither the sialylated (only), fucosylated (only), nor sialofucosylated species maintain the glycans across all samples uniformly. Closer inspection of the sites showed that AT1B2 site N118 and site N238 were two examples of sites that contain high mannose in nearly all samples. AT1B2 is a functionally important molecule at synapses as part of the ion pump Na^+^/K^+^ ATPase. Earlier studies have shown that 80% of its glycans are high mannose (2, 47). Another high mannose site that was found occupied by the same glycan type across nearly all samples was ADA10 (site N278). Further inspections of the heat maps showed that sialylated (only) and fucosylated (only) sites were preserved in some regions but not consistently as with high mannose type. Interestingly, when focusing on all the glycopeptides containing fucose-only (Fig. 9*D*) N-glycans, the signal transduction–type glycoproteins show high relative abundances in comparison to other molecular functions such as transport-type glycoproteins. However, the fucosylation is not preserved among all subjects. This is of importance as such processes are known to be mediated by cell surface glycosylation, and potential perturbation could result in a dysfunctional cell membrane. Sialofucosylated compositions were much more common across many regions and some subjects. For example, AT1B1 (site N158) maintained sialofucosylated glycans among nearly all regions and subjects. Interestingly, sialofucosylated N-glycans were most abundant on glycoproteins corresponding to regulatory processes as seen in Figure 9*E*. The glycan maps further showed the breadth of variations in the glycosites across different regions associated with different pathological conditions.

We note the limitations of this study, which is the small number of subjects prohibiting any conclusions regarding glycan-specific clinical indicators. However, these brain samples are difficult to obtain, but efforts are underway to increase the number of subjects. To increase the sample size further, we plan to focus the collection to a limited number of brain regions. The pilot study only involved male brains. Other limitations include the lack of comprehensive structural analysis of the glycans. The linkages, for example, are important component of the structures and strongly affect the function and specificity of the glycan interactions. The lack of glycan standards also prohibits absolute quantitation; however, the low coefficient of variations in the measurements allow accurate relative quantitation. Other limitations to address are the lack of transcriptional data and complete histopathological evaluation of samples.

In summary, our study demonstrated the most comprehensive glycan map of the brain of elderly adult males and showed that protein glycosylation could be altered by disease conditions. Some glycosites may be more affected than others, whereas glycosites containing high mannose–type structures were more persistent and less variable compared with other glycoforms. While the biological significance of the glycomic variation in the regions needs to be further resolved, the glycans appear to be primarily sialofucosylated species that are multiantennary with a high degree of branching. Sialic acids have been the primary focus of brain glycosylation, but the large abundance of fucosylation indicates that fucose should also be considered of great importance. The results further showed the broad compositional ranges of glycans between regions and between subjects suggesting potential targets for biomarkers and therapeutics.

## Data Availability

Mass spectrometry data for glycomics (https://doi.org/10.25345/C5686D, MSV000088628) and glycoproteomics (https://doi.org/10.25345/C52G5V, MSV000088629) are available on MassIVE data repository upon reasonable request.

## Supplemental data

This article contains supplemental data.
