# Supplementary material for: Regio-Specific N-Glycome and N-Glycoproteome Map of the Elderly Human Brain With and Without Alzheimer’s Disease
Source: Mol Cell Proteomics. 2022 Oct 14;21(11):100427. doi: 10.1016/j.mcpro.2022.100427 (PMC9674923; doi:10.1016/j.mcpro.2022.100427)
Supplement: Supplemental data 1 [file mmc1.docx]

Supplementary Materials for

**Regio-Specific N-Glycome and N-Glycoproteome Map of the Elderly Human Brain with and without Alzheimer’s Disease**

Jennyfer Tena,^1^ Izumi Maezawa,^2,3^ Mariana Barboza,^1,4^ Maurice Wong,^1^ Chenghao Zhu,^5^ Michael Russelle Alvarez,^6^ Lee-Way Jin,^2,3^ Angela M. Zivkovic^5^ and Carlito B. Lebrilla^1*^

*Corresponding author. Email: [cblebrilla@ucdavis.edu](mailto:xxxxx@xxxx.xxx)

**This PDF file includes:**

Supplementary Text

Figs. S1 to S6

Tables S1

Supplementary Text

Fig. S1A.

N-Glycome of the Occipital Cortex


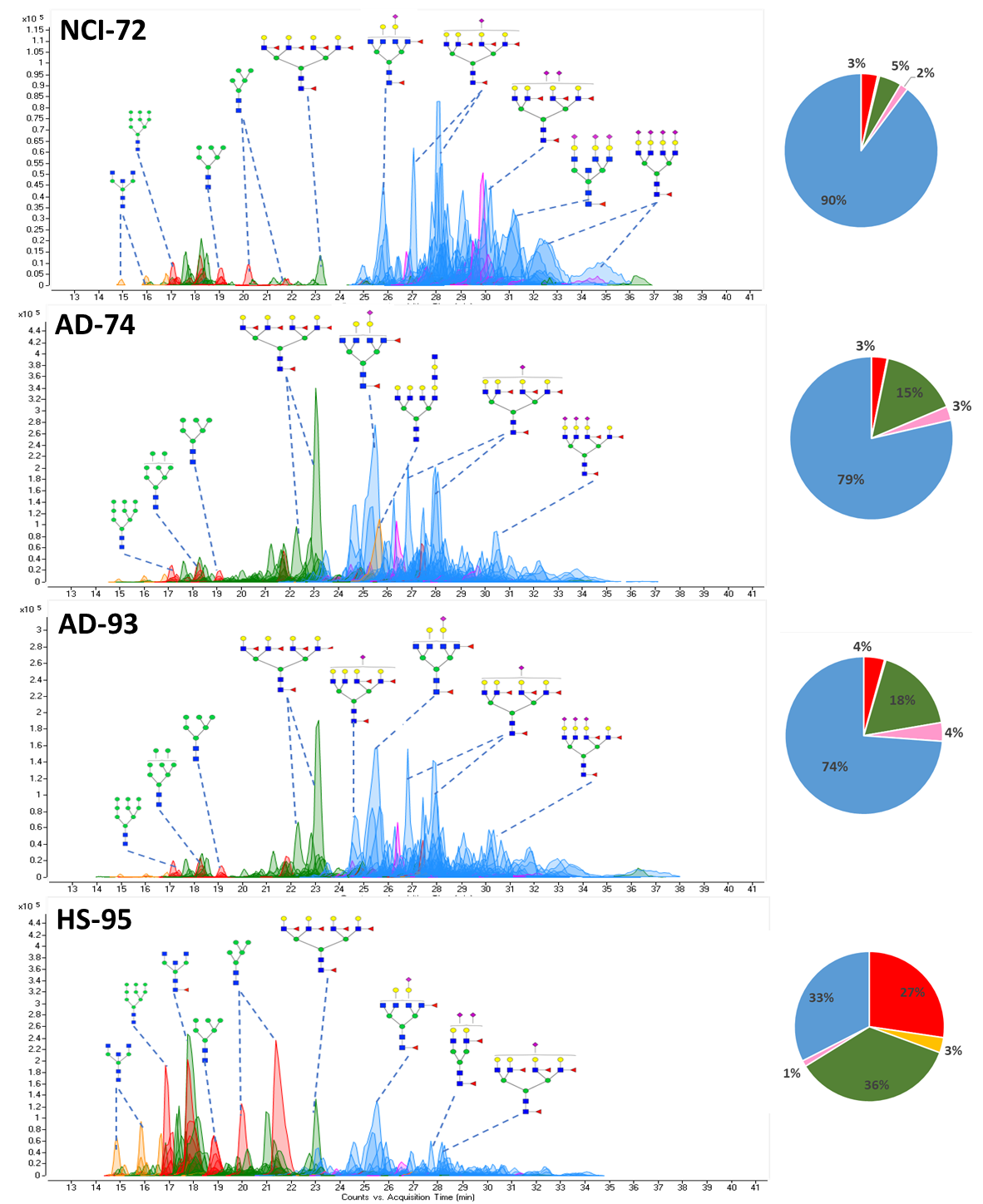


Fig. S1B.

N-Glycome of the Frontal Cortex.


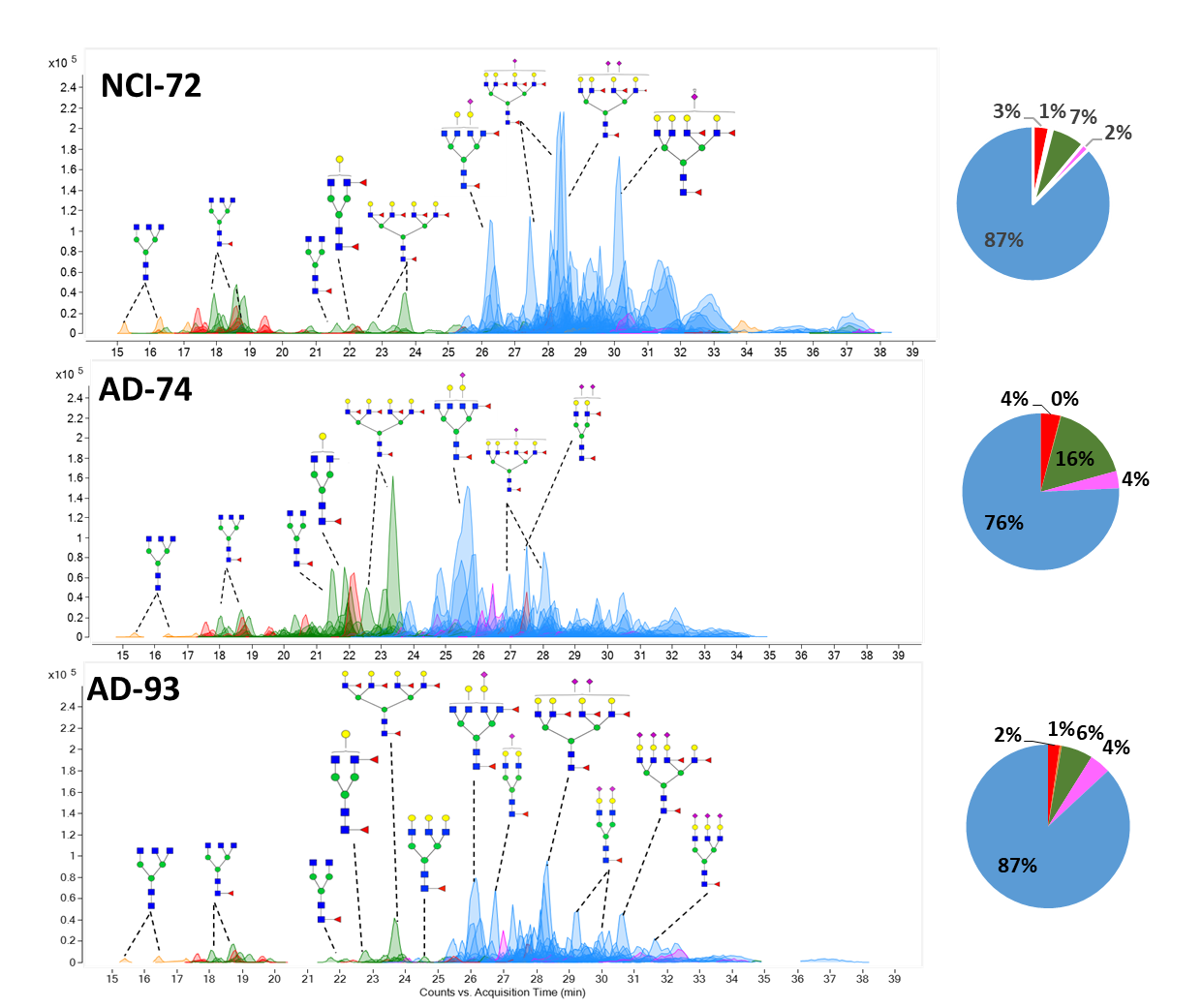


Fig. S1C.

N-Glycome of the Temporal Cortex.


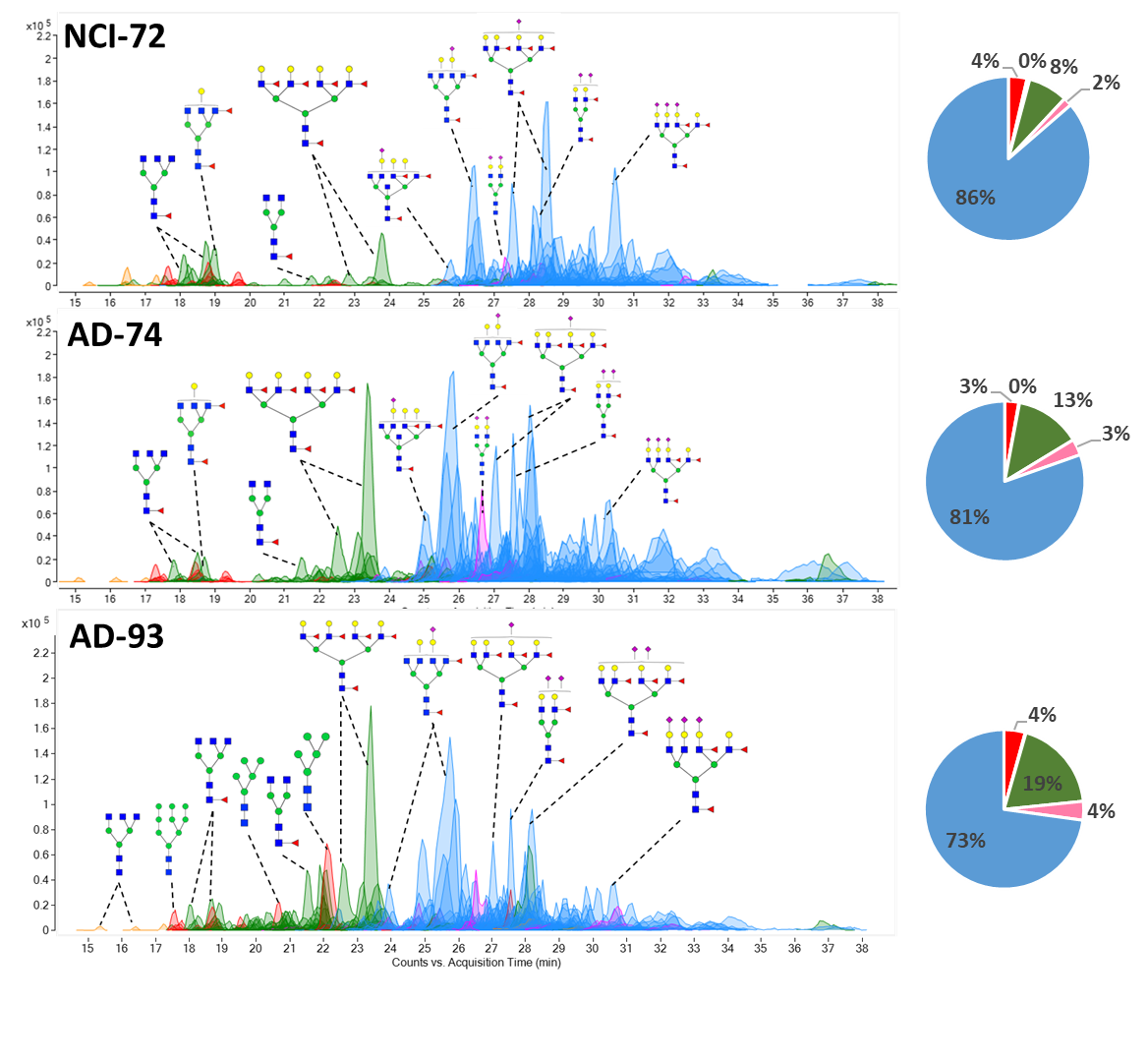


Fig. S1D.

N-Glycome of the Parietal Cortex.


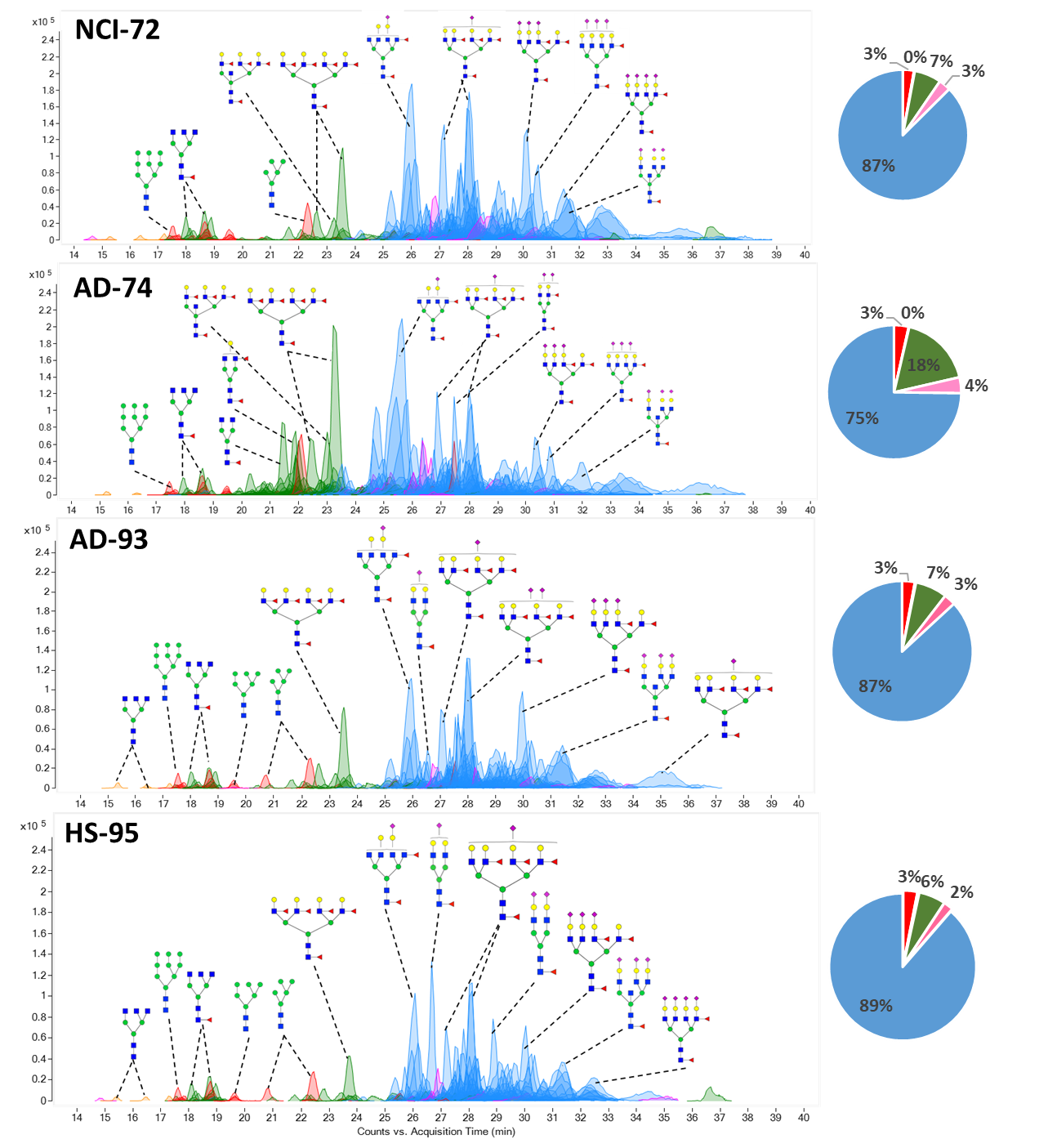


Fig. S1E.

N-Glycome of the Cingulate Cortex.


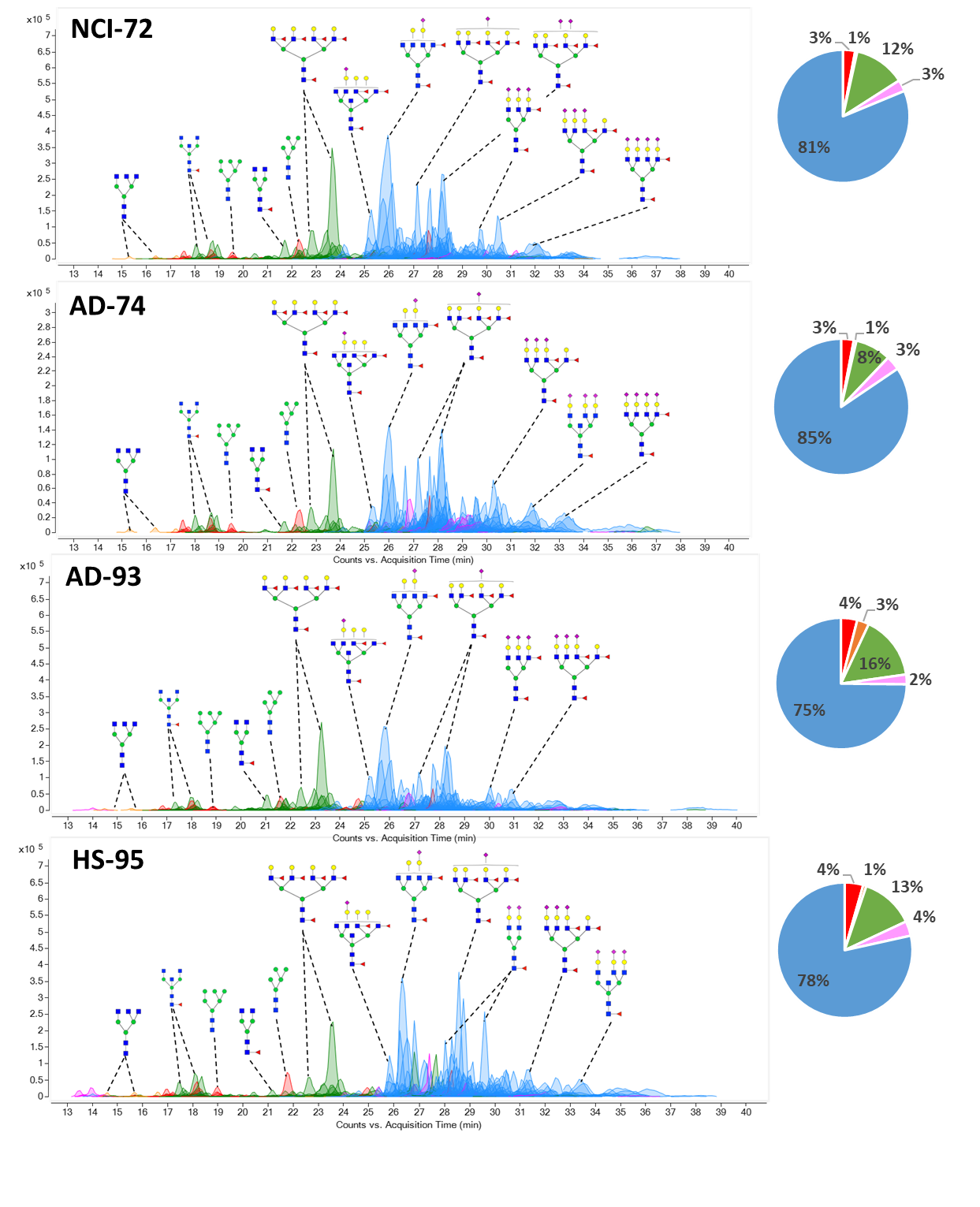


Fig. S1F.

N-Glycome of the Orbitofrontal Cortex.


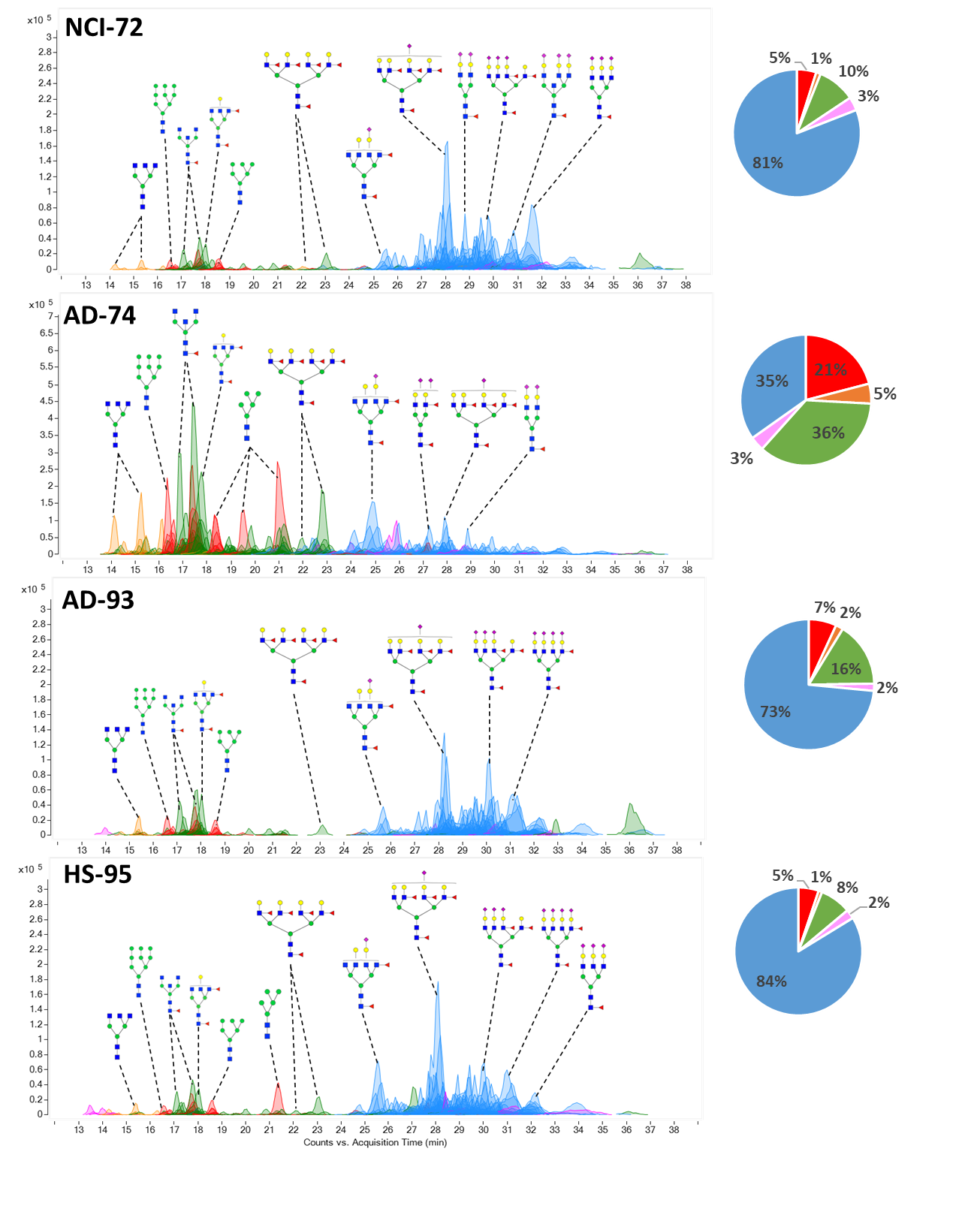


Fig. S1G.

N-Glycome of the Posterior Hippocampus.


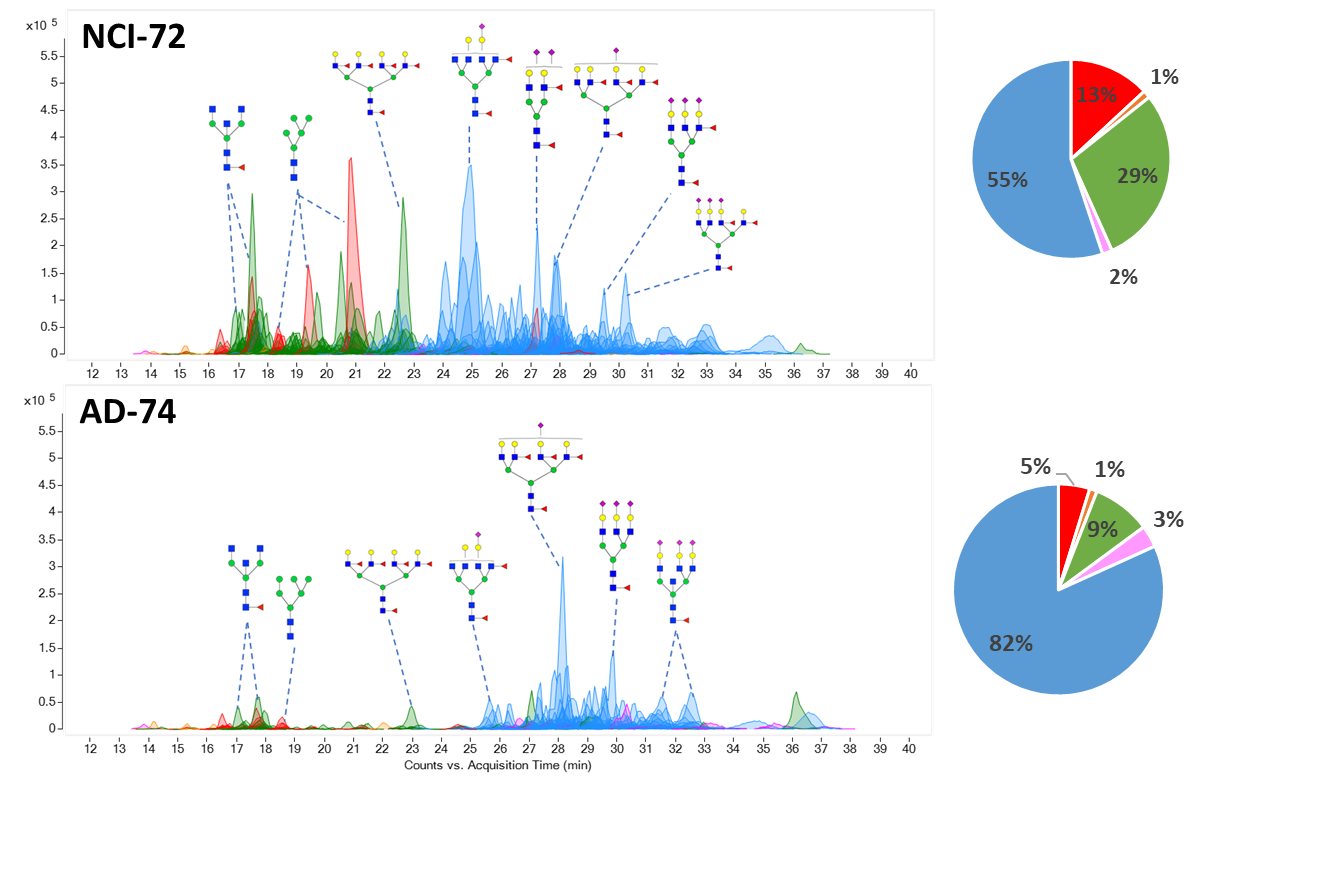


Fig. S1H.

N-Glycome of the Thalamus.


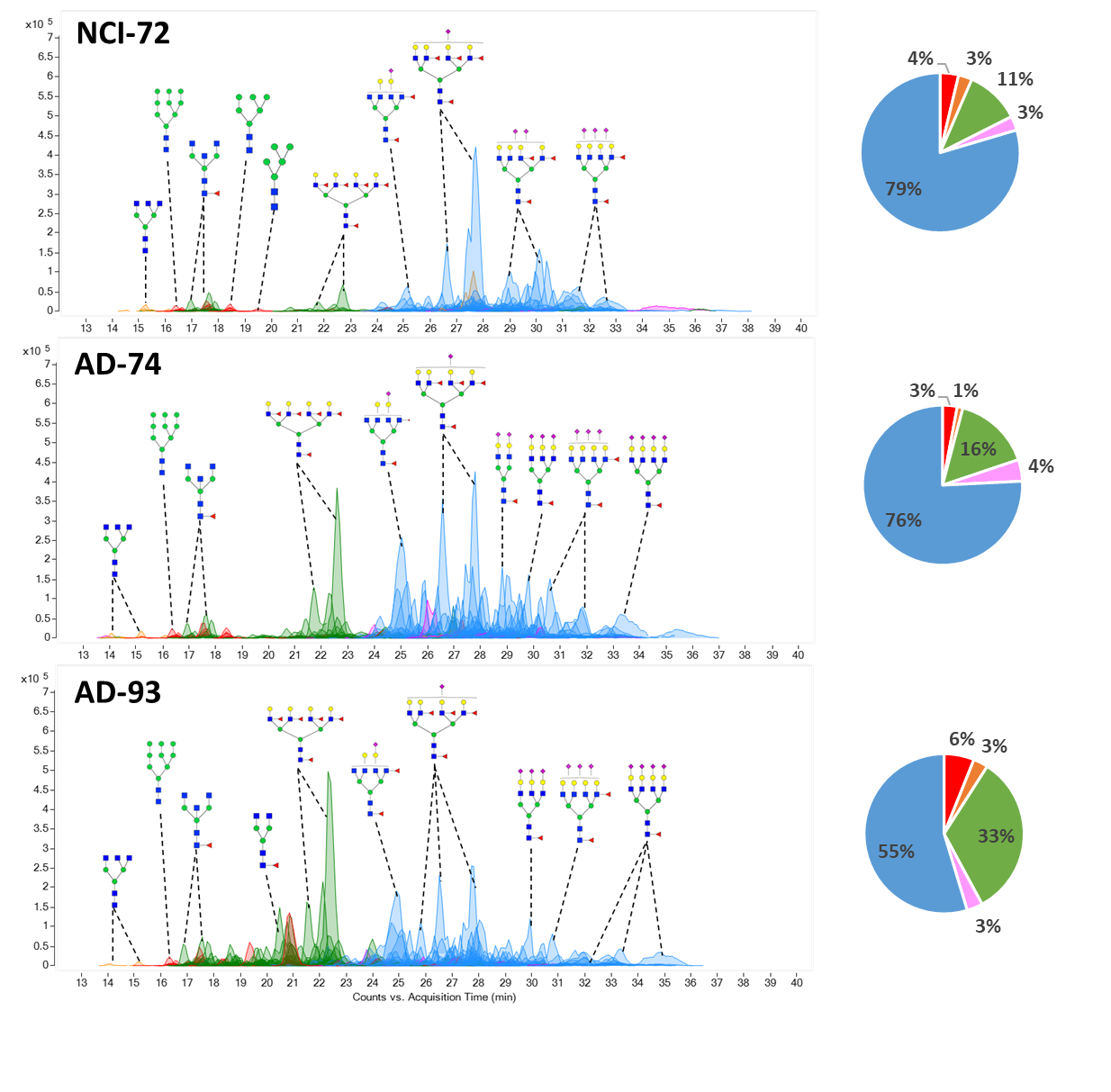


Fig. S1I.

N-Glycome of the Caudate Nucleus.


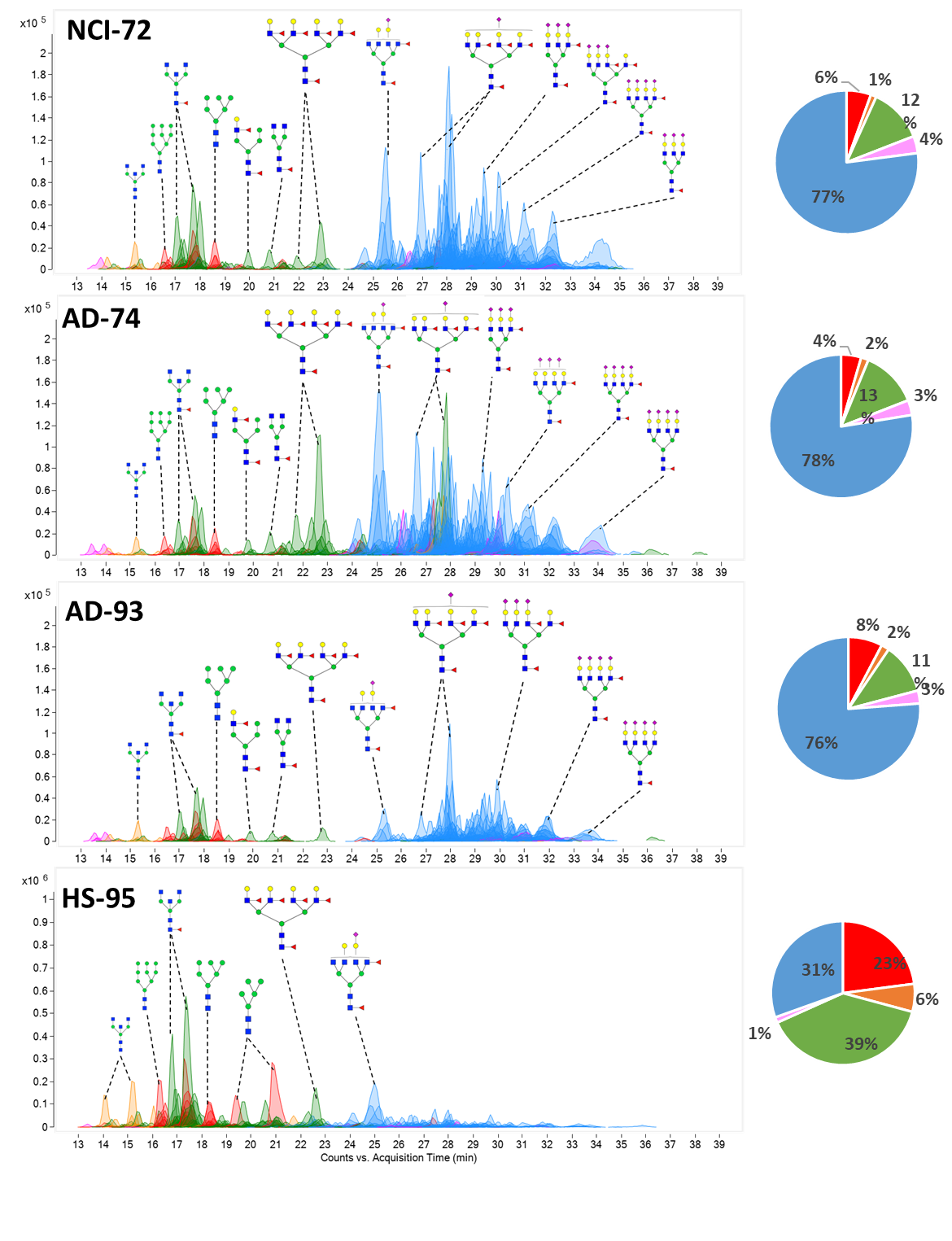


Fig. S1J.

N-Glycome of the Lateral Cerebellar Cortex.


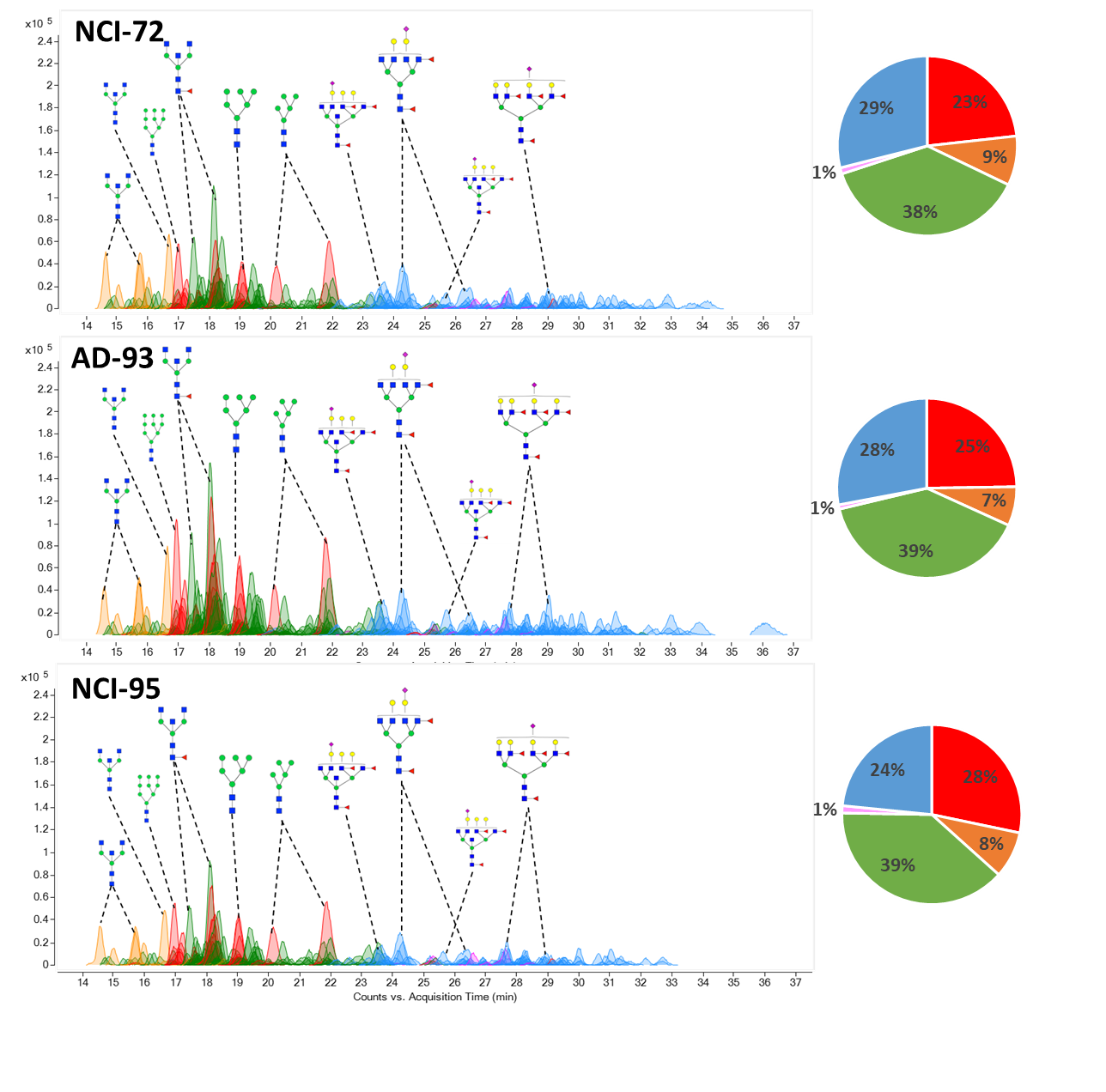


Fig. S1K.

N-Glycome of the Pons.


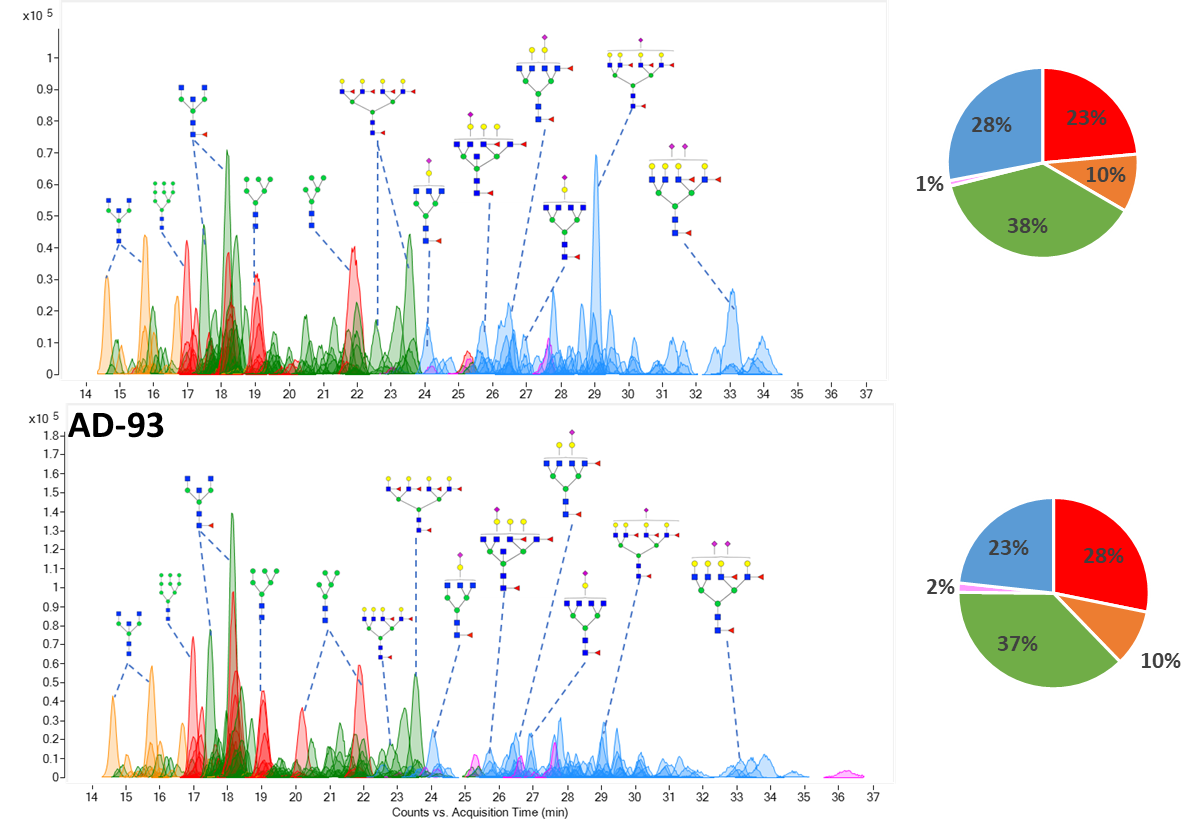


Fig. S2.

N-Glycans detected including isomers.


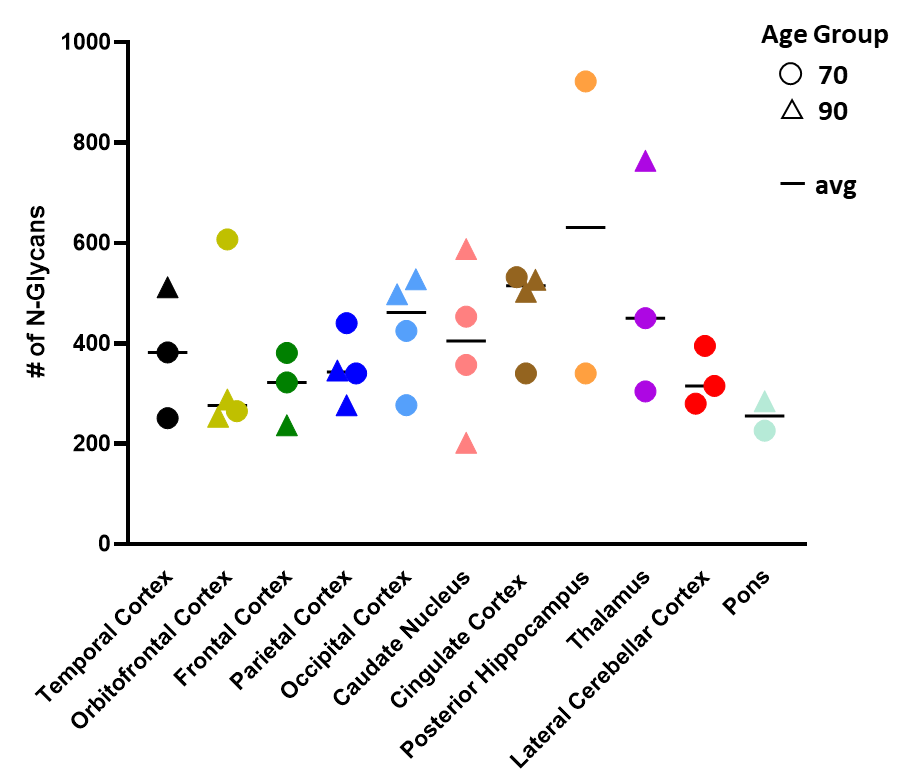


Fig. S3.

Structural analysis of N-glycans across all 11 brain regions.


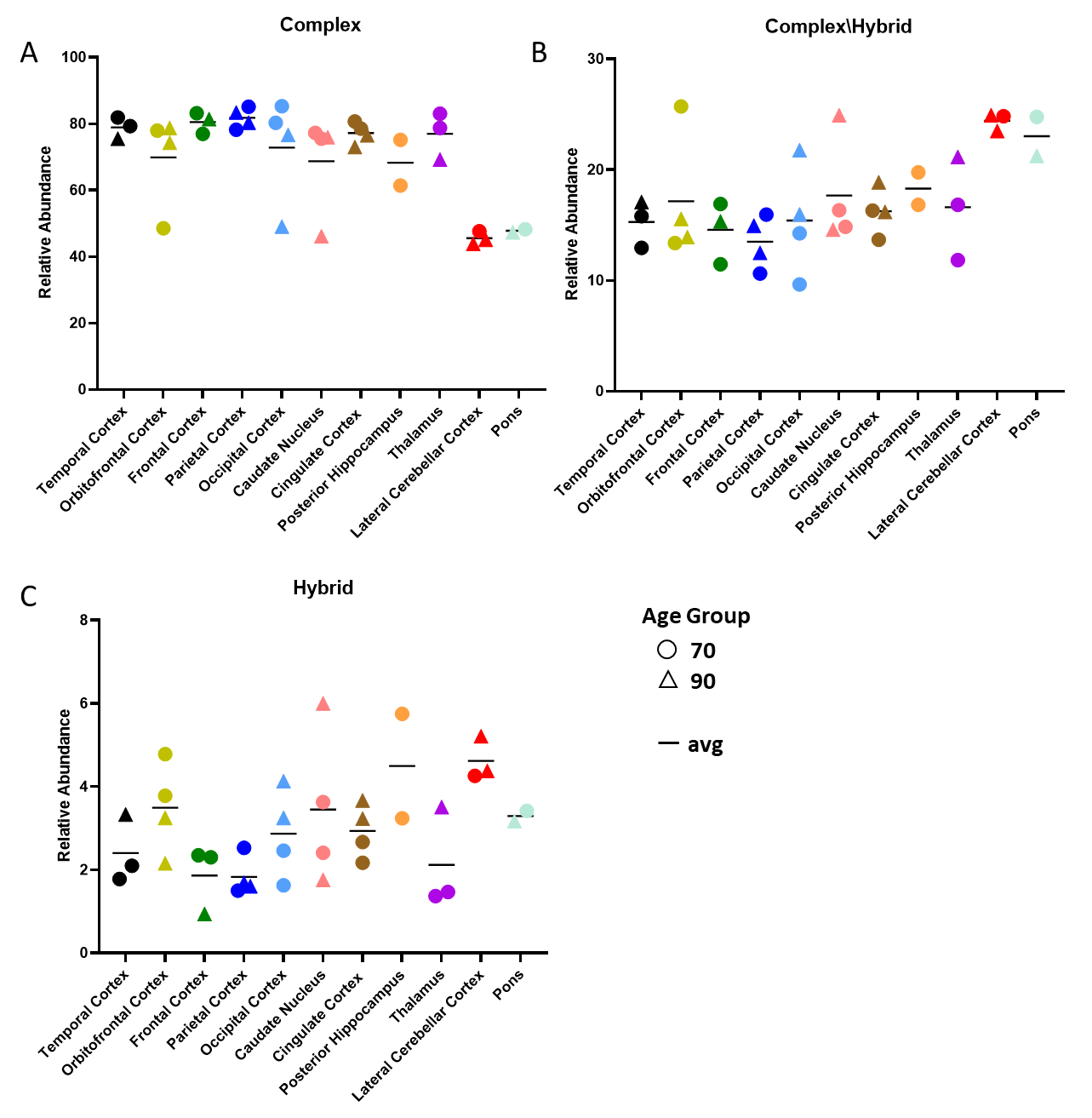


Fig. S4.

Heat map of the elderly human brain across 11 functional brain regions from four subjects.


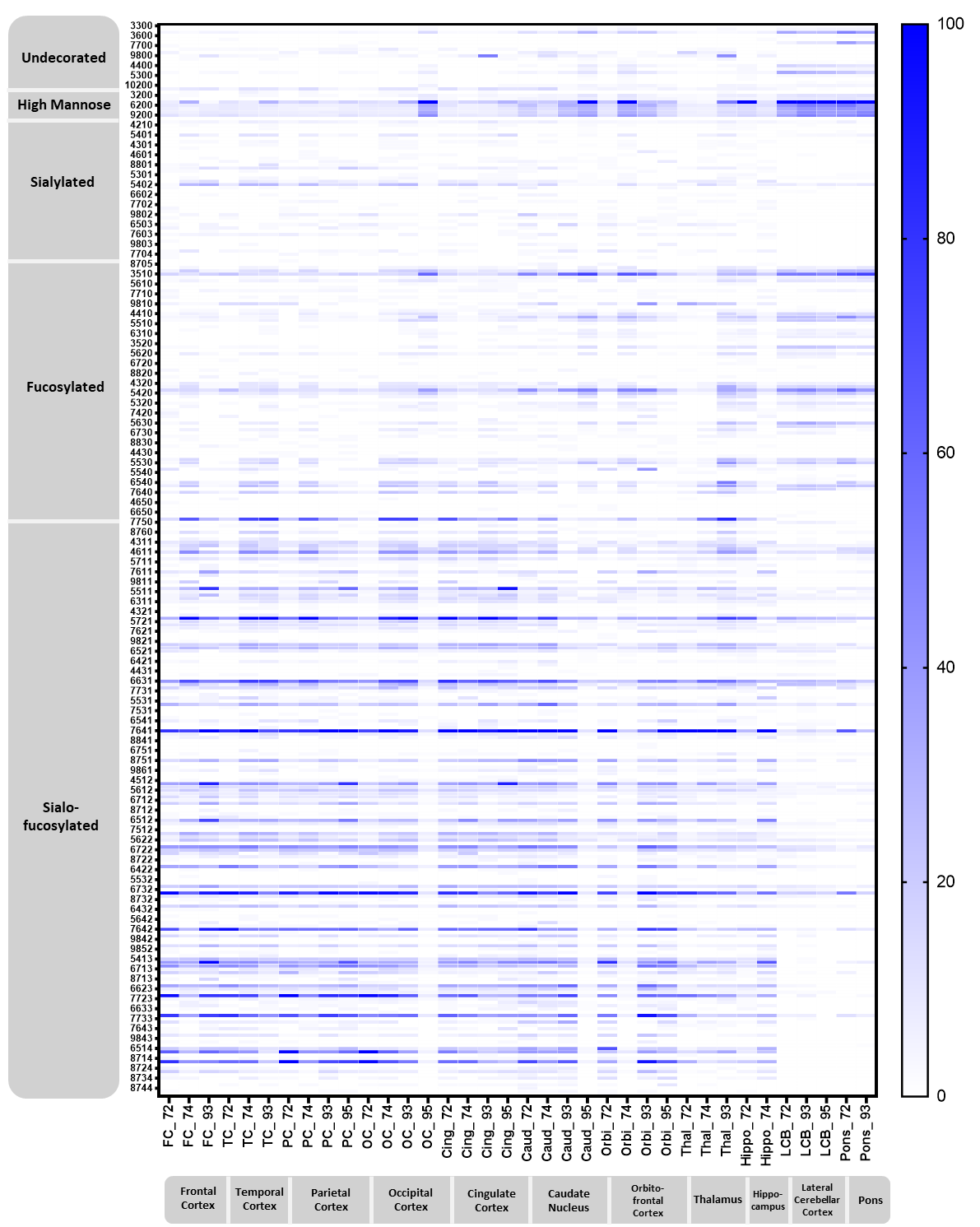


Fig. S5.

Region comparison based on N-glycan expression. Principal component analysis plot of human brain samples between 11 brains regions.


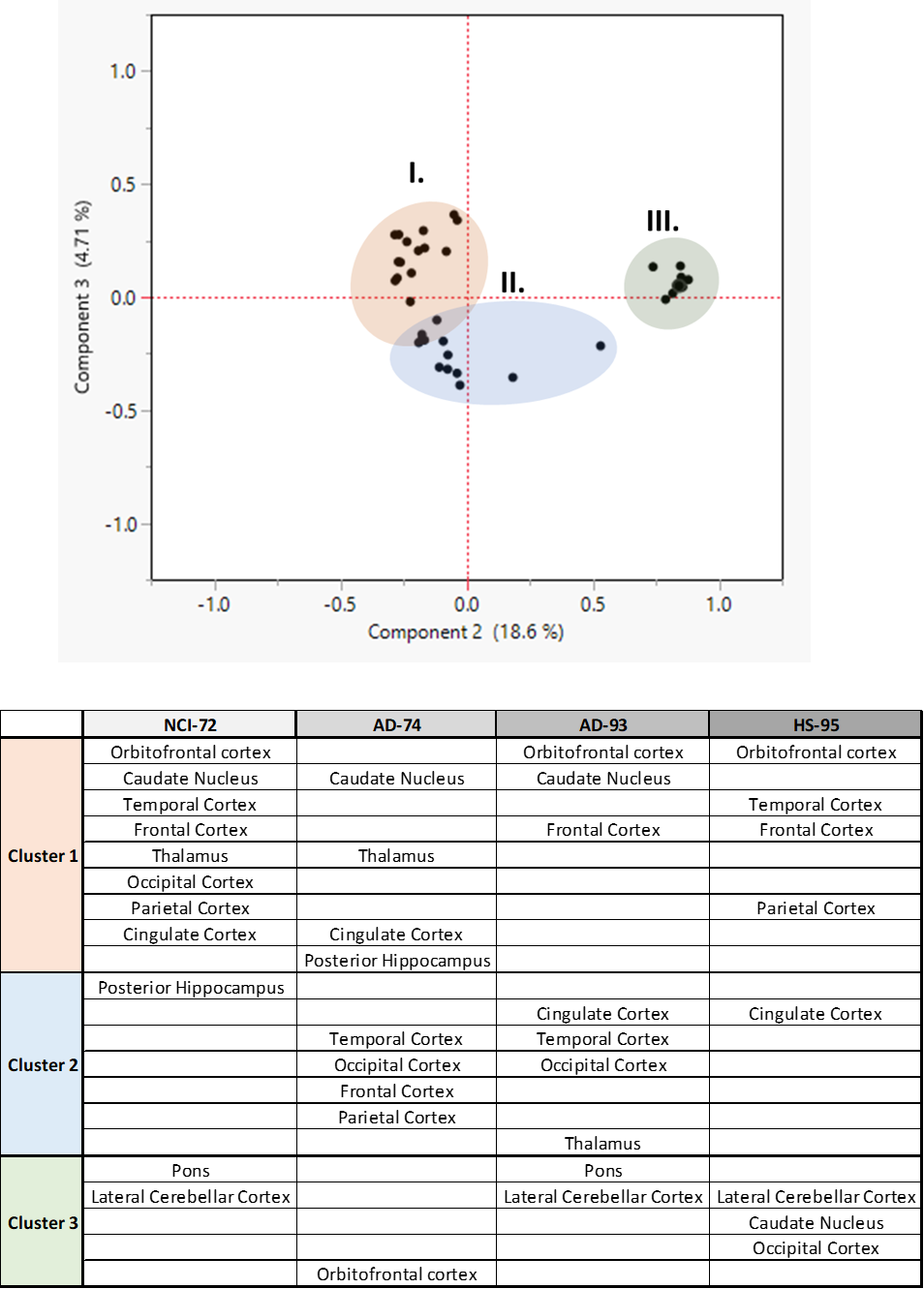


Fig. S6.

Comparison of the total membrane glycoproteins in the different regions.


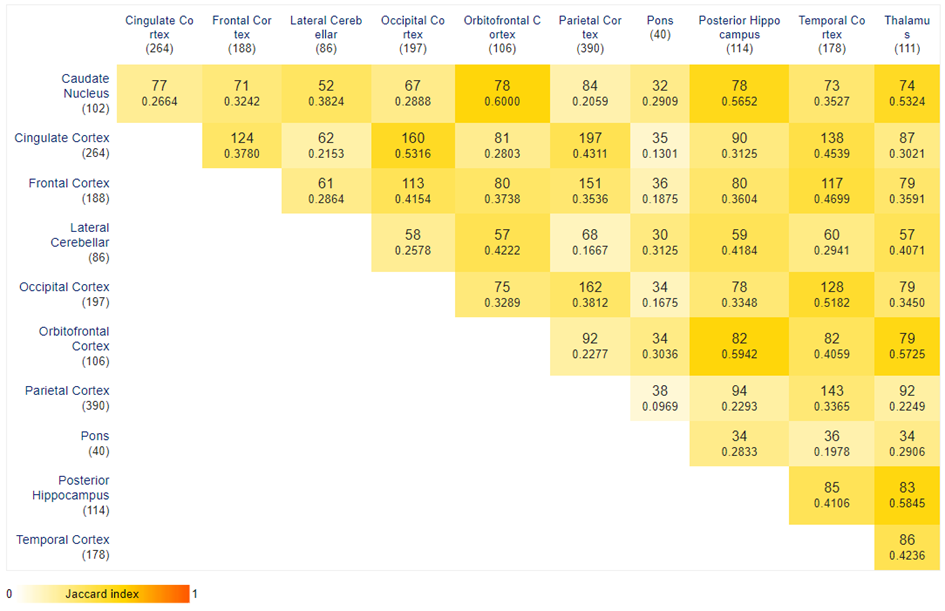


Table S1.

Human brain subjects utilized in this study for both control, AD and HS samples across eleven different brain regions.
